# Automated Screening of Microtubule Growth Dynamics Identifies MARK2 as a Regulator of Leading Edge Microtubules Downstream of Rac1 in Migrating Cells

**DOI:** 10.1371/journal.pone.0041413

**Published:** 2012-07-24

**Authors:** Yukako Nishimura, Kathryn Applegate, Michael W. Davidson, Gaudenz Danuser, Clare M. Waterman

**Affiliations:** 1 Cell Biology and Physiology Center, National Heart Lung and Blood Institute, National Institutes of Health, Bethesda, Maryland, United States of America; 2 Department of Cell Biology, The Scripps Research Institute, La Jolla, California, United States of America; 3 National High Magnetic Field Laboratory, Florida State University, Tallahassee Florida, United States of America; 4 Department of Cell Biology, Harvard Medical School, Boston, Massachusetts, United States of America; NCMLS, Radboud University Nijmegen Medical Center, The Netherlands

## Abstract

Polarized microtubule (MT) growth in the leading edge is critical to directed cell migration, and is mediated by Rac1 GTPase. To find downstream targets of Rac1 that affect MT assembly dynamics, we performed an RNAi screen of 23 MT binding and regulatory factors and identified RNAi treatments that suppressed changes in MT dynamics induced by constitutively activated Rac1. By analyzing fluorescent EB3 dynamics with automated tracking, we found that RNAi treatments targeting p150*^glued^*, APC2, spastin, EB1, Op18, or MARK2 blocked Rac1-mediated MT growth in lamellipodia. MARK2 was the only protein whose RNAi targeting additionally suppressed Rac1 effects on MT orientation in lamellipodia, and thus became the focus of further study. We show that GFP-MARK2 rescued effects of MARK2 depletion on MT growth lifetime and orientation, and GFP-MARK2 localized in lamellipodia in a Rac1-activity-dependent manner. In a wound-edge motility assay, MARK2-depleted cells failed to polarize their centrosomes or exhibit oriented MT growth in the leading edge, and displayed defects in directional cell migration. Thus, automated image analysis of MT assembly dynamics identified MARK2 as a target regulated downstream of Rac1 that promotes oriented MT growth in the leading edge to mediate directed cell migration.

## Introduction

Directed cell migration is fundamental to diverse physiological processes, including developmental morphogenesis, cancer metastasis, and the immune response. To directionally migrate, cells must sense signals from the extracellular environment and transduce the signals to downstream effectors that polarize the cytoskeleton and regionally regulate its organization and dynamics [1]. It is well established that the actin and microtubule (MT) cytoskeletal systems are critical to directed cell movement. The actin cytoskeleton is the primary force generator for cell migration: Rapid polymerization of F-actin drives lamellipodia/lamella protrusions in the cell leading edge, and contraction of acto-myosin bundles in the cell body causes retraction of the trailing cell rear [2].

The MT cytoskeleton is required for rapid, directed migration of tissue cells [3], however its role in cell motility is less clear than that of actin. MTs are thought to provide critical cell polarization cues that dictate spatial regulation of protrusive and contractile actin-based processes [3]. Indeed, the MT cytoskeleton displays a characteristic polarized organization and dynamics in migrating cells. MTs growing from the MT-organizing center (MTOC) and Golgi apparatus orient towards the leading edge lamella/lamellipodia region [3], [4]. In addition, MT dynamic instability is polarized in migrating cells. MTs in the lamellipodia grow more slowly, persistently and parallel to the cell edge than those in the lamella [5], [6], [7]. These leading edge MTs have been termed “pioneer MTs,” and in PtK epithelial cells, exhibit a decreased catastrophe frequency (switch from growth to shrinkage), resulting in an increased growth time [7]. Pioneer MTs are thought to guide directed migration by localizing signals that promote lamellipodial actin dynamics [8], and/or facilitating turnover of leading edge focal adhesions [9]. However, the mechanisms that establish pioneer MTs in the lamellipodia of migrating cells are not understood.

The Rho family of small GTPases act as molecular switches that locally control cytoskeleton dynamics through multiple effectors [10]. In migrating cells, activated Rac1 accumulates in lamellipodia and stimulates local actin polymerization to drive leading edge protrusion [11], [12]. In addition, we previously showed that lamellipodial pioneer MTs can be induced by activation of Rac1 [7]. We identified two distinct pathways downstream of Rac1 that promote pioneer MT growth. One depends on Rac1-mediated activation of p21-activated kinase1 (Pak1), which phosphorylates and inactivates the MT-destabilizing protein Op18/stathmin [7], [13]. Alternatively, Rac1-mediated inactivation of glycogen-synthase kinase 3β (GSK3β) induces MT binding and growth stabilization by CLASP [14], [15]. However, in those studies, we found that neither Pak1 activation nor GSK3β inhibition were sufficient for inducing pioneer MTs [7], [13], [15], indicating that additional factors must regulate pioneer MTs downstream of Rac1.

To find downstream targets of Rac1 signaling that promote pioneer MTs, we performed an RNAi-based screen of MT regulatory factors to identify proteins whose depletion suppresses MT growth and orientation induced by constitutively activated Rac1. For unbiased and statistically robust analysis of millions of MT growth excursions in many cells across a range of conditions (Tables S1, S3, S5, S7, S8), we utilized plusTipTracker, an automated image analysis program that tracks motion of fluorescent-tagged EB3, a MT plus-end binding protein (+TIP) [16], [17], [18]. We identified six RNAi treatments that suppressed Rac1-induced MT assembly dynamics, and one of these, microtubule-affinity-regulating kinase2 (MARK2), that additionally suppressed Rac1-mediated MT orientation in lamellipodia. The MARK2 homologues are known to mediate organization of MTs and polarity establishment in epithelia, oocytes and neurons [19], [20], [21], [22], [23], but how MARK2 controls MT dynamics in cell migration has not been demonstrated. Our results suggest that MARK2 is required for polarization in migrating cells through promotion of pioneer MTs downstream of Rac1.

## Results

### Rac1 Activity Promotes Pioneer MTs in Lamellipodia of U2-OS Osteosarcoma Cells

**Figure 1:**
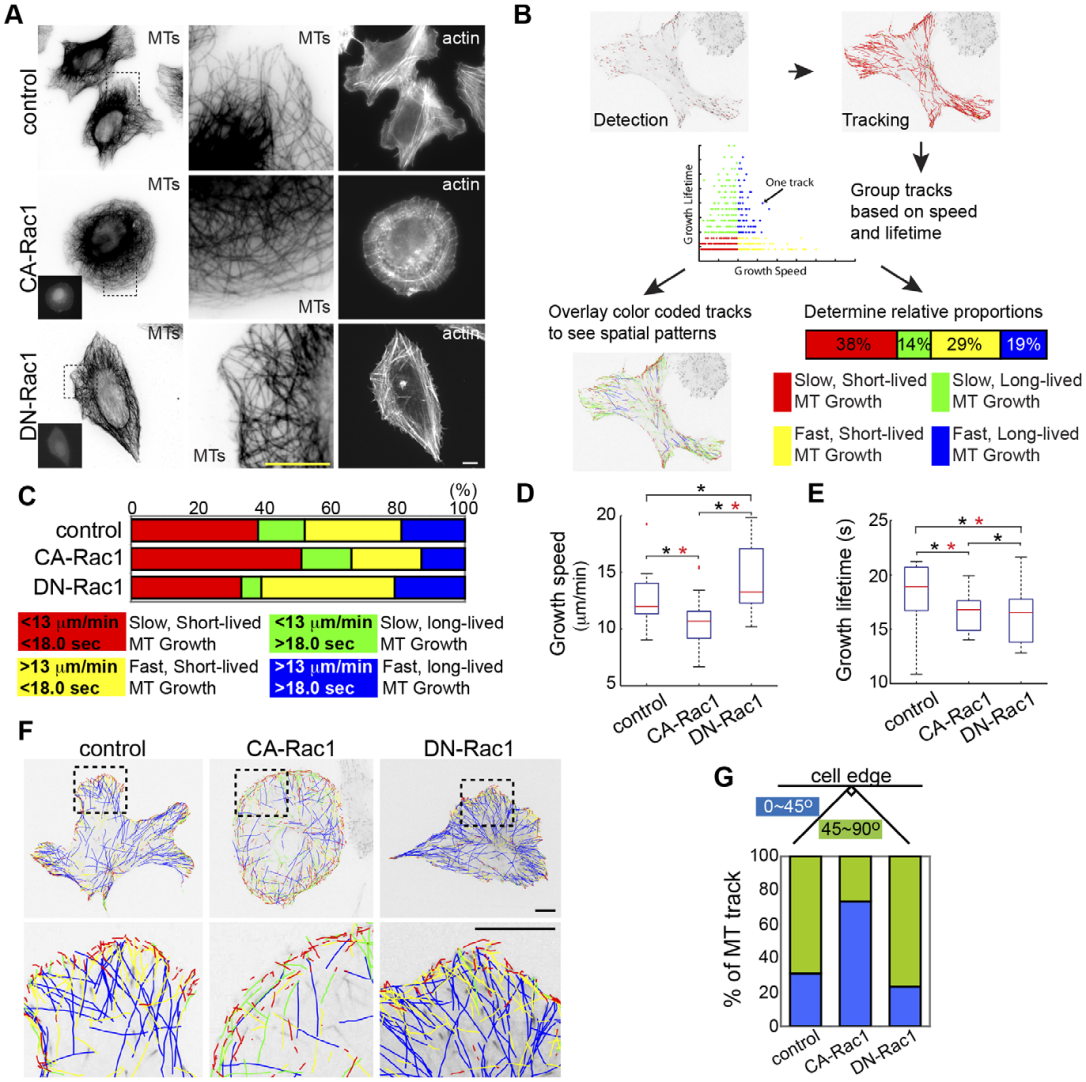
Rac1 promotes pioneer MTs in U2-OS cells. (A) Immunolocalization of microtubules (MTs) and fluorescent phalloidin staining of F-actin in mock-transfected cells (control) or cells expressing CA-Rac1 or DN-Rac1. Contrast inverted, left, center. Zoom of boxed region, second column. Insets, BFP-Rac1. Bar, 10 µm. (B) Workflow of plusTipTracker software for detecting mKO-EB3 comets, tracking them, and classifying MT growth excursions. (C) Top: Proportion of MT growth excursions in each subpopulation in non-targeting control vector, CA-Rac1 or DN-Rac1 expressing cells. Bottom: Color key showing MT growth speed and growth excursion lifetime ranges for subpopulations. Box-plots of speed (D) and lifetime (E) of MT growth excursions, conditions as in C. (*, p<0.001, Kolmogorov-Smirnov, *, p<0.05, Students). (F) Top: mKO-EB3 tracks from 2 min time-lapse movies (frame rate  = 3 s) colored according to the key in C overlaid on images of mKO-EB3 (inverted contrast), conditions described in A. Bottom: zoom of boxed region. Bars, 10 µm. (G) Percentage of MT growth tracks within 5 µm from the leading edge whose angle relative to the edge is between 0–45° (blue) or between 45–90° (green), conditions as in C.

We previously showed that Rac1 activation in PtK1 kidney epithelial cells promotes formation of pioneer MTs, characterized by persistent, slow plus-end growth in lamellipodia parallel to the leading cell edge [7]. To identify downstream targets of Rac1 that promote pioneer MTs, we sought to utilize an RNAi screening approach with U2-OS osteosarcoma cells due to their human origin and amenability to transfection. To validate the efficacy of these cells for our purpose, we transfected them with a constitutively activated Rac1 mutant (CA-Rac1) and analyzed their MT organization and dynamics. Fluorescent localization of F-actin showed that expression of CA-Rac1 in U2-OS cells promoted lamellipodia formation and reduced stress fibers compared to mock-transfected controls or cells expressing dominant negative Rac1 (DN-Rac1) (Fig. 1A) and similar to effects in other cell types [24]. Immunolocalization of MTs showed that like previous studies in PtK1 cells, expression of CA-Rac1 had a strong effect on MT organization, inducing a bundle of MTs parallel to the cell edge in lamellipodia around most of the cell periphery. In contrast, in controls and cells expressing DN-Rac1, MTs were for the most part oriented perpendicular to the cell edge and absent from lamellipodia. Thus, Rac1 activity promotes formation of oriented lamellipodial MTs in U2OS cells, similar other cell types [7].

To determine the effects of Rac1 activity on MT growth dynamics, we performed live-cell imaging of the +TIP protein EB3 fused to monomeric Kusabira Orange (mKO-EB3, Video S1) as a non-perturbing marker of growing MT plus ends [25], [26] and used plusTipTracker software package [16], [17], [18] for high-throughput measurement of MT growth dynamics. Because +TIP proteins only associate with MT ends during active growth excursions, we were limited to measuring MT growth speed and growth persistence lifetime, i.e. the duration of a growth excursion before loss of fluorescent EB3 from the MT plus end due to transition to shortening (catastrophe) or to pause [25]. Automated tracking is powerful because of the ability to measure ∼10,000–30,000 MT growth excursions per condition (Tables S1, S3, S5, S8), allowing robust statistical analysis that facilitates identification of treatments that may have subtle, yet reproducible effects on MT growth. The limitations and accuracy of this program are described in detail in [16], [17].

We classified MT growth excursions as “slow” vs. “fast” or “short-lived” vs. “long-lived” based on thresholds of the mean value for each parameter from the population of all measurements from non-targeting vector-transfected control cells (Fig. 1B, [17], [18]). These classes of MT growth dynamics were color-coded and the percentage of growth excursions in each class in the measured population was depicted as a bar graph to allow easy visual assessment and comparison of the effects of perturbations on MT growth dynamics across a range of conditions [17], [18]. Mean growth speeds and growth lifetimes, standard errors and the number of cells and MTs analyzed, for all conditions in all figures are presented in the Supplemental tables. We also generated image overlays of color-coded MT growth tracks to allow qualitative visualization of regional differences in MT growth dynamics throughout the cell (Fig. 1B).

Analysis of MT growth with plusTipTracker software showed that compared to control, CA-Rac1 expression increased the proportion of slow, short-lived (red) MT growth excursions, whereas expression of DN-Rac1 increased the proportion of fast, short-lived (yellow) MT growth excursions (Fig. 1C, Table S2). Changes in proportions of MTs in each class were mirrored by changes in mean values, as CA-Rac1 decreased and DN-Rac1 increased MT growth speed, and either CA-Rac1 or DN-Rac1 reduced MT growth lifetime (Fig. 1D, 1E, Table S1) compared to control. These results differ from previous results where CA-Rac1 was shown to inhibit catastrophe and thus promote longer-lived MT growth [7]. This discrepancy could arise from the technical improvements afforded by our current methodology including automated tracking of EB3 comets compared to hand-tracking fluorescent tubulin in our previous studies [5], [7]. These improvements include a shorter image acquisition frequency, which would allow measurement of shorter-lived growth excursions than the previous study, and higher accuracy localization of the MT end, which would allow measurement of slower growth that may have been counted as pause in the previous study. Moreover, manual measurements of MT dynamics tend to focus on longer-lived growth phases. These subjective biases are reduced by automated tracking approaches. Alternatively, Rac1 activity may promote subtle differences in MT dynamic instability depending on cell type. Nonetheless, the overall phenotype of CA-Rac1 expression, with extended MTs running parallel to the cell edge in the lamellipodia are similar in both studies, indicating that Rac1 activity generates major effects on MTs in multiple cell types.

We next examined the spatial distribution of MT growth induced by CA-Rac1. Given that pioneer MTs grow parallel to the leading edge [5], we classified the orientation of MT growth tracks within 5 µm of the cell edge as “parallel” (0–45°) or “perpendicular” (45–90°, Fig. 1G, Table S7). This analysis and examination of image overlays of color-coded MT growth tracks (Fig. 1F) showed that CA-Rac1 promoted slow MT growth (red and green) in the cell periphery parallel to the cell edge, and long-lived MT growth (blue and green) in the cell center. In contrast, controls and cells expressing DN-Rac1 displayed fast, long-lived MT growth (blue) in the cell center, and both fast and slow, short-lived MT growth (red and yellow) perpendicular to the leading edge at the cell periphery. Thus, our results show that activated Rac1 mediates pioneer MT formation in U2-OS cells at least in part by promoting slow, short-lived MT growth excursions in lamellipodia parallel to the cell edge, thus allowing for the basis of a phenotypic screen.

### An RNAi Screen Identifies Proteins that Modulate MT Dynamics Downstream of Rac1 in U2-OS Osteosarcoma Cells

**Figure 2:**
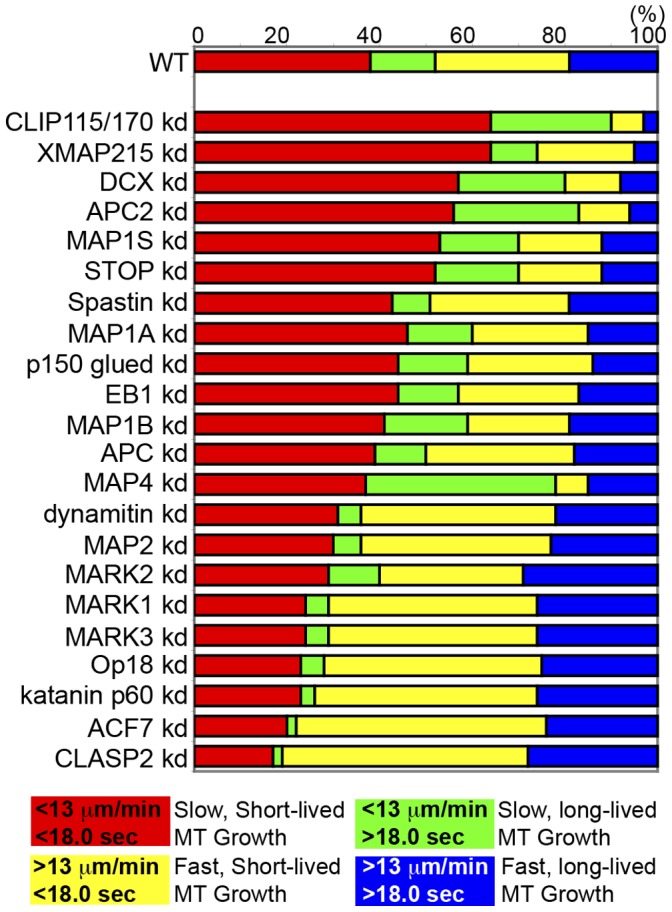
Effects of MT regulatory protein depletion on MT growth dynamics in U2-OS cells. Top: Results of analysis of time-lapse movies of mKO-EB3 with PlusTipTracker software. Proportion of MT growth excursions in each subpopulation in control shRNA vector-transfected cells (WT) or cells treated with RNAis targeting the protein noted (kd). shRNA vectors were used for RNAi targeting of EB1, CLASP2, dynamitin, DCX, MAP1A, MAP1B, MAP2, MAP4, MARK1, MARK2 and MARK3. siRNA oligos were used for RNAi targeting of APC, APC2, ACF7, XMAP215, Op18, p150*^glued^*, CLIP115, CLIP170, STOP, MAP1S, Spastin and Katanin p60 (see Methods). Bottom: Color key showing MT growth speed and growth excursion lifetime ranges for each subpopulation.

**Figure 3:**
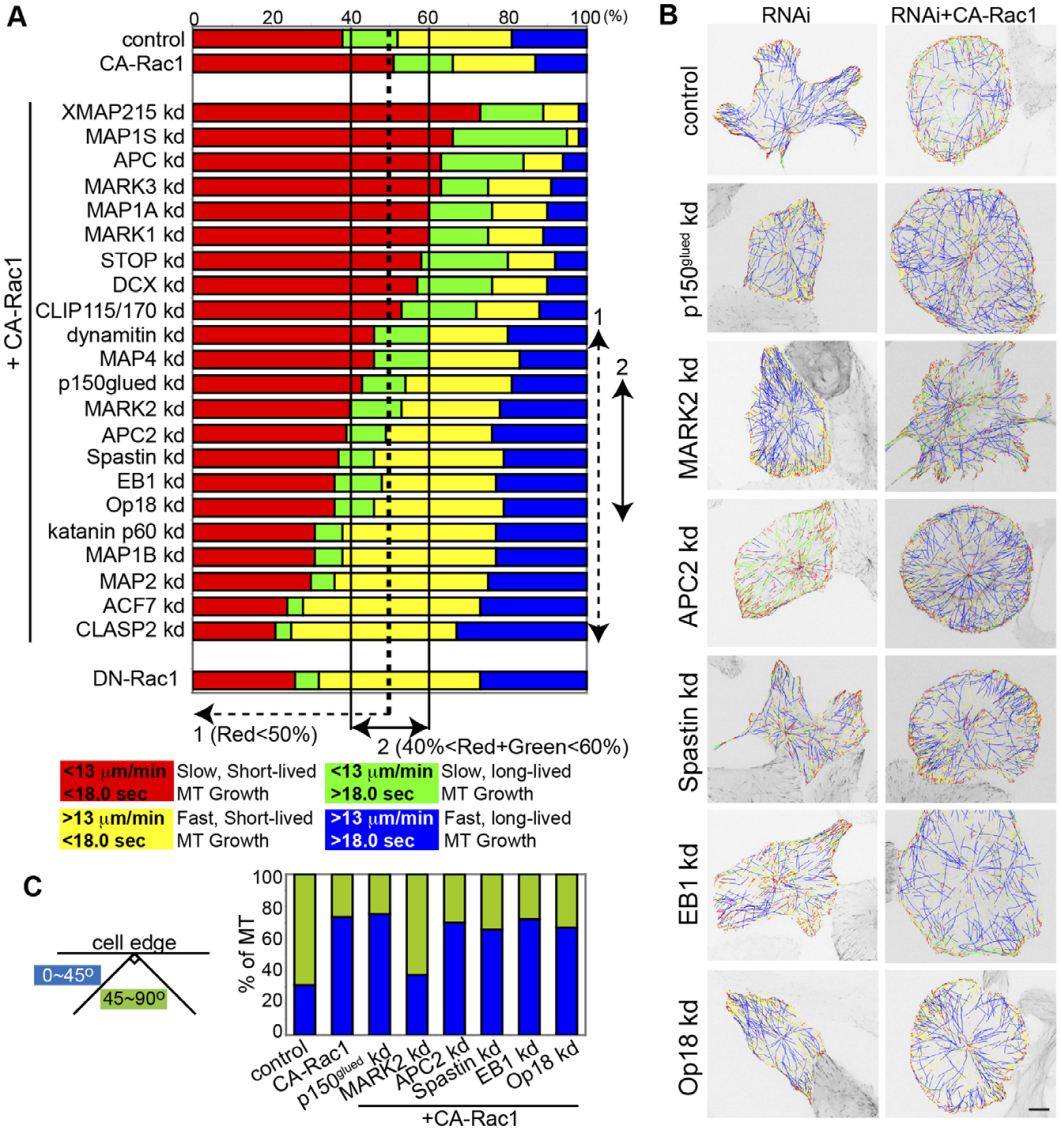
RNAi screen for proteins whose depletion blocks CA-Rac1 effects on MT growth and orientation. (A) Top: Proportion of MT growth excursions in each subpopulation in control shRNA vector-transfected cells (control), cells expressing CA-Rac1, CA-Rac1 and additionally treated with RNAis targeting the protein noted (kd), DN-Rac1. shRNA vectors were used for RNAi targeting of EB1, CLASP2, dynamitin, DCX, MAP1A, MAP1B, MAP2, MAP4, MARK1, MARK2 and MARK3. siRNA oligos were used for RNAi targeting of APC, APC2, ACF7, XMAP215, Op18, p150*^glued^*, CLIP115, CLIP170, STOP, MAP1S, Spastin and Katanin p60 (see Methods). Dashed line 1: proteins which pass the first criteria (red tracks<50%); solid line 2: proteins which pass the second criteria (40%<red+green tracks <60%). Bottom: Color key showing MT growth speed and growth excursion lifetime ranges for subpopulations. (B) mKO-EB3 tracks from 2 min time-lapse movies (frame rate = 3 s) colored according to the key in A overlaid on images of mKO-EB3 (inverted contrast) in cells treated with RNAis targeting the noted proteins with (bottom) or without (top) CA-Rac1 expression. Bar, 10 µm. (C) Percentage of MT growth tracks within 5 µm from the leading edge whose angle relative to the cell edge is between 0–45° (blue) or between 45–90° (green), conditions as in B.

To find downstream targets of Rac1 that promote pioneer MTs, we performed RNAi-based depletion of 23 MT-regulatory factors (Figs. 2 and 3) and screened for proteins whose depletion suppresses the slow, short-lived MT growth parallel to the leading edge induced by CA-Rac1 in U2-OS cells (Fig. 3). We focused our screen on non-motor MT-binding target proteins that are known to regulate MT growth behavior, but it is unknown if they are regulated by Rac1. These included APC, APC2, ACF7, CLASP2, XMAP215, CLIP170/115, p150*^glued^*, p50/dynamitin, doublecortin, EB1, katanin p60 subunit, MARK1, MARK2, MARK3, MAP1A, MAP1B, MAP1S, MAP2, MAP4, STOP, spastin, or Op18/stathmin. shRNA vectors were used for RNAi targeting of EB1, CLASP2, dynamitin, DCX, MAP1A, MAP1B, MAP2, MAP4, MARK1, MARK2 and MARK3. siRNA oligos were used for RNAi targeting of APC, APC2, ACF7, XMAP215, Op18, p150*^glued^*, CLIP115, CLIP170, STOP, MAP1S, spastin and katanin p60 (see Methods). Op18/stathmin and CLASP that are both known to regulate MT dynamics downstream of Rac1 in other cell types [13], [15] were included as positive controls. We used plusTipTracker to analyze the speed, lifetime, and orientation of MT growth excursions in movies of cells expressing mKO-EB3 and treated with either siRNAs or shRNAs targeting (see Methods) one of these MT regulatory proteins together with (Fig. 3A, Tables S5, S6) or without (Fig. 2, Tables S3, S4) the additional expression of CA-Rac1. Expression of target proteins in U2OS cells was verified for as many target proteins as possible (see methods), however in many cases antibodies were not available. Thus, negative results in this screen should be interpreted with caution.

We first analyzed the effects of RNAi treatments targeting MT regulatory proteins in the absence of CA-Rac1. As expected, RNAi treatment targeting most of these MT regulatory proteins induced alterations of MT growth behavior compared to non-targeting RNAi controls (Fig. 2, see Methods). RNAi treatments targeting either the +TIP rescue factor CLIP115/170 [27], the XMAP215 protein that promotes rapid MT assembly and disassembly [28], the MT stabilizer and assembly factor DCX [29], the plus end anchor and MT stabilizing protein APC2 [30], [31], the MT growth enhancer MAP1S [32] or the MT stabilizer STOP [33] resulted in a greater fraction of both slow short-lived (red) and slow long-lived (green) MT growth excursions compared to control. This suggests that these proteins promote fast MT growth in U2-OS cells, and confirms demonstrated (XMAP215 [28], DCX [29]) and suggested (STOP [33], MAP1S [32], APC2 [34]) functions of several of these proteins from previous studies in other cell types. RNAi treatments targeting either the dynactin subunit p50/dynamitin, the MT stabilizer MAP2, the microtubule affinity regulating kinases (MARK1, MARK2, MARK3) that promote MT disassembly [35], the catastrophe factor Op18/stathmin [36], the MT severing protein katanin p60 [37], the MT-actin crosslinking and catastrophe-promoting protein ACF7 [38], or the MT growth stabilizer CLASP2 [39] resulted in a greater fraction of both fast short-lived (yellow) and fast long-lived (blue) MT growth excursions compared to control. This suggests that these proteins slow or inhibit MT growth in U2-OS cells, and confirms demonstrated (Op18/stathmin [36], katanin [37], ACF7 [38]) and suggested (MARKs [35], CLASP [39]) functions of these proteins from previous studies in other cell types. RNAi treatments targeting each of the remaining proteins (the +TIP EB1, the MT severing protein Spastin, the assembly promoting MAP1 family members (MAP1A and MAP1B), the dynactin subunit p150*^glued^*, and the growth-promoting +TIP APC had little effect on the rate or duration of MT growth excursions, suggesting possibly inefficient RNAi targeting, low protein expression in U2-OS cells, the presence of redundant proteins (i.e. APC2, EB3, MAP1s), or cell type-specific effects. Differences in expected effects of RNAi treatment on MT dynamics based on previous studies (i.e. CLIP115/170, MAP2) could be due to similar issues with RNAi targeting and/or cell type specificity. However, in general, these results demonstrate that depletion of known MT regulatory proteins produces expected effects on U2-OS MT growth dynamics.

To identify proteins whose depletion suppresses the slow, short-lived MT growth parallel to the leading edge induced by CA-Rac1, we then determined the effects of RNAi treatments targeting MT regulatory proteins in the presence of activated Rac1. We considered a “hit” based on three criteria. First, because the percentage of slow, short-lived (red) MT growth tracks was increased in cells expressing CA-Rac1 (Fig. 1C, 51%) compared to controls (38%), we sought targets whose RNAi treatment reduced the percentage of slow, short-lived (red) MT growth tracks in cells expressing CA-Rac1 to less than 50% (black dashed line, Fig. 3A). RNAi treatments meeting this criterion included those targeting dynactin subunits (p50/dynamitin, p150*^glued^*), MAPs (MAP4, MAP1B, MAP2), MT severing proteins (Spastin, katanin p60), MT plus end-binding proteins (EB1, APC2, CLASP2), the MT depolymerizing factor Op18/stathmin, the cytoskeletal linker ACF7, and MARK2 (Table S6). Our identification of Op18 and CLASP, previously known targets of Rac1 regulation [13], [15], validates the efficacy of our automated MT analysis and selection criteria approach. Second, because CA-Rac1 increased the total proportion of slow-growing MTs (red+green, 65%) compared to control (red+green, 52%), our second criteria for a “hit” was an RNAi treatment that reduced the percentage of slow growth tracks (red+green) in cells expressing CA-Rac1 to between 40% and 60% (black lines, Fig. 3A). RNAi treatments meeting both criteria included those targeting p150*^glued^*, APC2, Spastin, EB1, Op18, and MARK2 (Table S6), suggesting that these proteins are required for the slow, short-lived MT growth induced by CA-Rac1.

As a third criterion, we next sought to identify target proteins whose RNAi targeting additionally suppressed the effects of CA-Rac1 on MT orientation in the lamellipodium. We analyzed image overlays of color-coded MT growth tracks in cells expressing CA-Rac1 and treated with siRNAs or shRNAs targeting p150*^glued^*, APC2, spastin, EB1, Op18 and MARK2 (Fig. 3B, 3C, Table S7). Of these, only cells expressing CA-Rac1 and shRNA targeting MARK2 showed a reduction in MTs oriented parallel to the leading edge compared to cells expressing CA-Rac1 alone. Together, these results suggest that MARK2 may be a key Rac1 target promoting leading edge pioneer MTs by regulating both MT growth speed and lifetime as well as growth orientation in the cell periphery.

### MARK2 Promotes Short-lived MT Growth Parallel to the Cell Edge Downstream of Rac1

**Figure 4:**
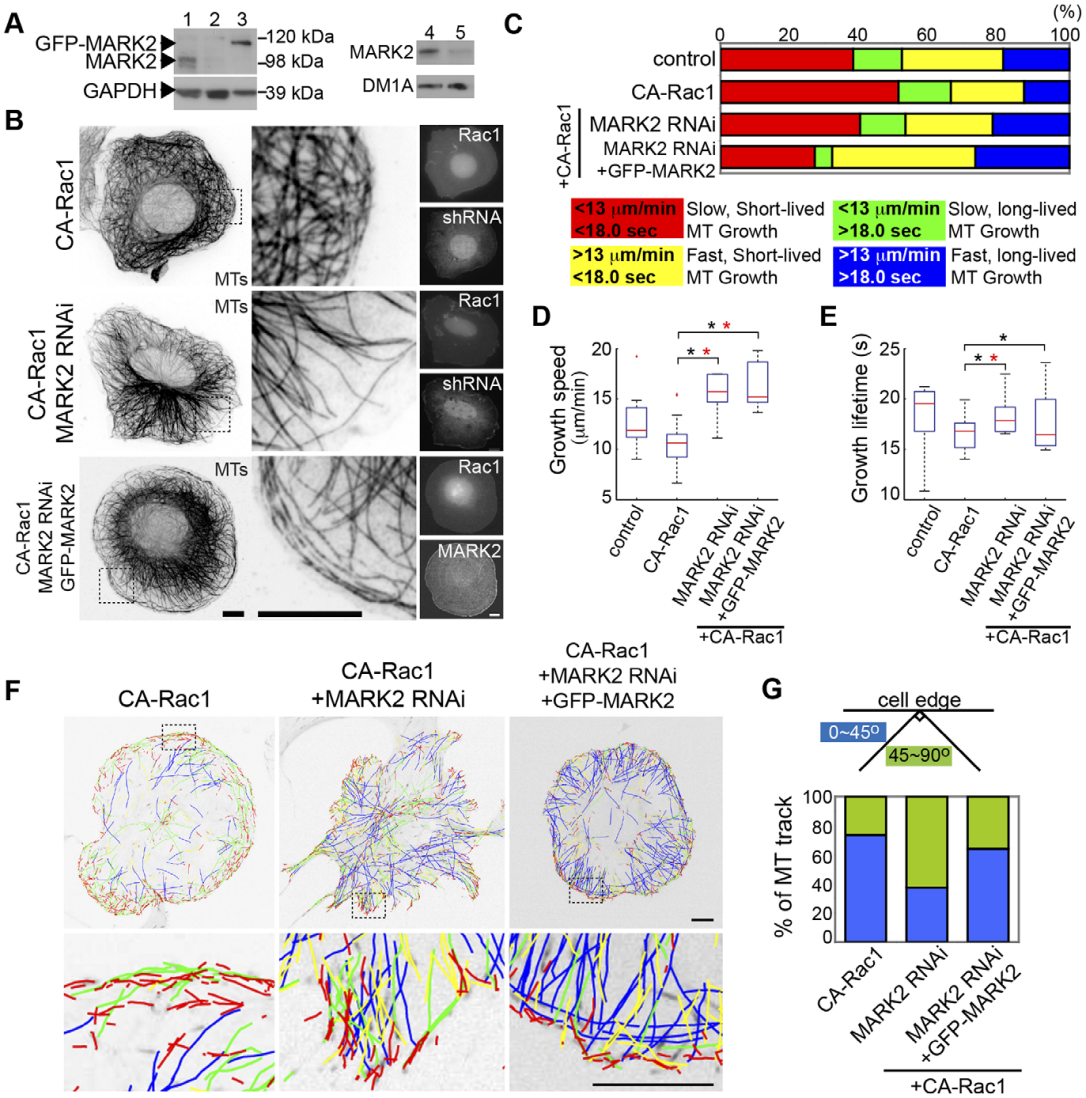
MARK2 regulates MT growth dynamics and orientation downstream of Rac1. (A) Western blot of lysates of U2-OS cells transfected with control shRNA (lane 1), MARK2-shRNA (lane 2), MARK2-shRNA and shRNA-resistant GFP-MARK2, (lane 3), non-targeting siRNA pool (lane 4) or MARK2 siRNA (lane 5). GAPDH and DM1A were used as a loading control. (B) Immunostaining of MTs (inverted contrast) in cells expressing BFP-CA-Rac1 (Rac1) and GFP-shRNA targeting MARK2 (shRNA), or rescued with GFP-MARK2 expression. Center column: zoom of boxed region. (C) Top: Proportion of MT growth excursions in subpopulations, conditions as in B. Control represents control shRNA vector transfected cells. Bottom: Color key showing MT growth speed and growth excursion lifetime ranges for subpopulations. Box-plots of speed (D) and lifetime (E) of MT growth excursions, conditions as in B. (*p<0.001, Kolmogorov-Smirnov, *p<0.05, Students). (F) Top: mKO-EB3 tracks from 2 min time-lapse movies (frame rate = 3 s) colored according to the key in C overlaid on images of mKO-EB3 (inverted contrast), conditions as in B. Bottom: Zoom of boxed regions. (G) Percentage of MT growth tracks within 5 µm from the leading edge whose angle relative to the cell edge is between 0–45° (blue) or between 45–90° (green), conditions as in B. Bars, 10 µm.

We next sought to validate the results of our RNAi screen suggesting that Rac1’s effects on MT dynamics and orientation are mediated by MARK2. Western blot of U2-OS cell lysates showed that either shRNA or siRNA effectively depleted MARK2 protein (Fig. 4A, lanes 2 and 5) and expression of an shRNA-resistant GFP-tagged MARK2 in cells expressing MARK2-specific shRNA restored near wild-type protein level (Fig. 4A, lane 3). In fixed cells, both the elongated MTs in lamellipodia parallel to the cell edge and the discoid cell morphology induced by CA-Rac1 were abolished by shRNA-mediated MARK2 depletion, and MT organization and cell morphology were rescued by re-expression of GFP-MARK2 (Fig. 4B). Analysis of MT growth dynamics via tracking mKO-EB3 (Video S2) showed that shRNA-mediated MARK2 depletion in cells expressing CA-Rac1 increased the proportion of fast, long-lived (blue) MT growth and decreased slow, short-lived (red) growth (Fig. 3A, 4C, Table S2), increasing mean MT growth speed and lifetime compared to cells expressing CA-Rac1 alone, and similar to these values in control shRNA treated-cells (Fig. 4D, 4E, Table S1). Expression of GFP-MARK2 in shRNA-mediated MARK2-depleted, CA-Rac1-expressing cells rescued the effects of MARK2 depletion on MT growth lifetime (Fig. 4E) and growth orientation in lamellipodia (Fig. 4F, 4G, Table S7). However, despite rescue of MT growth lifetime and orientation, expression of GFP-MARK-2 in MARK2 shRNA-treated cells further increased MT growth speed compared to cells expressing CA-Rac1 alone (Fig. 4D). To determine if the increased MT growth speed was due to an off-target effect, we tested to see if an shRNA targeting a distinct sequence in MARK2 had similar effects on MT dynamics ([40], Fig. S1, Tables S1 and S8). This showed that both shRNAs had similar effects: increases in both MT growth speed and lifetime, and expression of GFP-MARK2 rescued the increased MT growth lifetime but showed further increase of MT growth speed (Fig. 4D, 4E, Fig. S1, Table S8). This suggests that MT growth speed may be tightly regulated by a specific MARK2 protein level [41]. However, these results show that MARK2 promotes pioneer MT formation in lamellipodia downstream of activated Rac1, at least in part by inducing short-lived MT growth excursions parallel to the cell edge.

### MARK2 is Required for Pioneer MTs in the Leading Edge and Polarized Cell Migration

**Figure 5:**
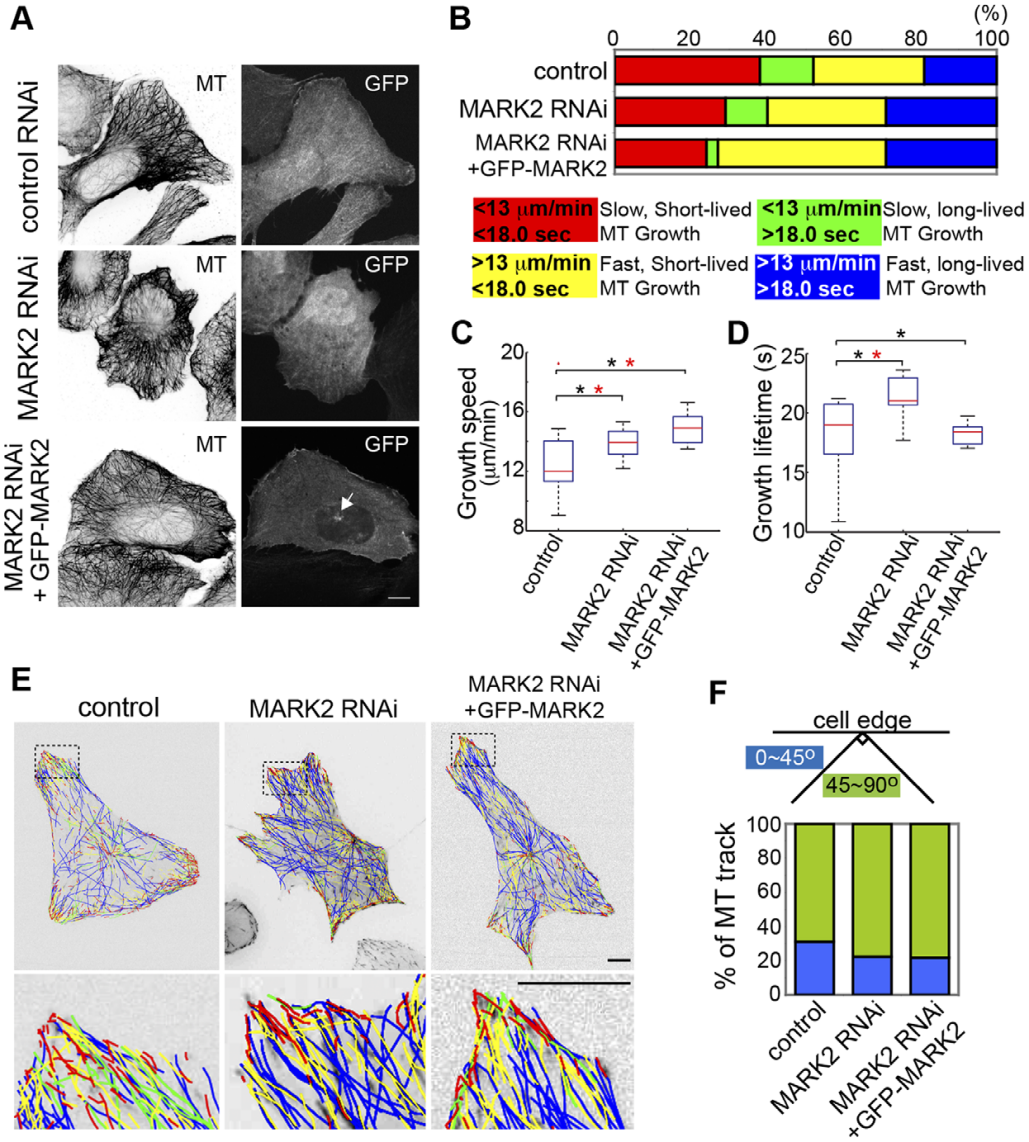
MARK2 regulates MT growth lifetime. (A) Immunolocalization of MTs (inverted contrast) in cells expressing control shRNA-GFP (top), MARK2 shRNA-GFP (middle), or MARK2 shRNA-GFP together with RNAi-resistant GFP-MARK2 (bottom). Cells co-expressing MARK2 shRNA-GFP and GFP-MARK2 could be recognized by the targeting of GFP-MARK2 to the centrosome (arrowhead). (B) Top: Proportion of MT growth excursions in each subpopulation in cells under the conditions described in A. Bottom: Color key showing MT growth speed and growth excursion lifetime ranges for subpopulations. Box-plots of speed (C) and lifetime (D) of MT growth excursions, conditions as in A. (*, p<0.001, Kolmogorov-Smirnov, *, p<0.05, Students) (E) Top: mKO-EB3 tracks from 2 min time-lapse movies (frame rate = 3 s) colored according to the key in B overlaid on images of mKO-EB3 (inverted contrast), conditions as in A. Bottom: zoom of boxed regions. (F) Percentage of MT growth tracks within 5 µm from the leading edge whose angle relative to the cell edge is between 0–45° (blue) or between 45–90° (green), conditions as in A. Bars, 10 µm.

We then characterized the function of MARK2 in U2OS cells, comparing its role in MT organization and dynamics in non-polarized and polarized migrating cells. Staining of MTs in fixed non-polarized cells showed that neither shRNA-mediated MARK2 depletion nor re-expression of GFP-MARK2 in MARK2-depleted cells substantially affected overall MT organization (Fig. 5A). However, analysis of MT growth dynamics via tracking mKO-EB3 (Video S2) showed that MARK2 depletion did, in fact, affect MT dynamics in non-polarized cells. As noted, MARK2 shRNA treatment increased the proportion of fast, long-lived MT growth excursions (blue) and reduced the proportion of both slow short-lived and slow long-lived growth excursions (red and green, Fig. 2, Fig. 5B, Table S1), increasing both speed and lifetime of MT growth (Fig. 5C, 5D, Table S2). Expression of GFP-MARK2 in MARK2-depleted non-polarized cells increased the proportion of fast, short-lived MT growth excursions (yellow), rescuing the effects of MARK2 depletion on MT growth lifetime, but further increased MT growth speed compared to control (Fig. 5B–D). This is similar to effects of rescuing MARK2 depletion with GFP-MARK2 in the presence of CA-Rac1 (Fig. 4D, 4E, Video S2), supporting the notion that RNAi effects on MT lifetime were MARK2-dependent and effects on growth speed may be dependent on the specific level of MARK2 protein or were MARK2 independent. Analysis of the spatial organization of MT growth excursions in image overlays of color-coded MT growth tracks showed that in non-polarized cells, neither shRNA-mediated MARK2 depletion nor re-expression of GFP-MARK2 in MARK2 depleted cells substantially affected the spatial distribution or orientation relative to the cell edge of MT growth excursions (Fig. 5E, 5F, Table S7). Thus, although MARK2 clearly regulates MT growth lifetime and orientation in lamellipodia downstream of activated Rac1, in non-polarized cells in the absence of exogenous Rac1 activation, MARK2 promotes short-lived MT growth excursions, but has little effect on MT organization or growth orientation.

**Figure 6:**
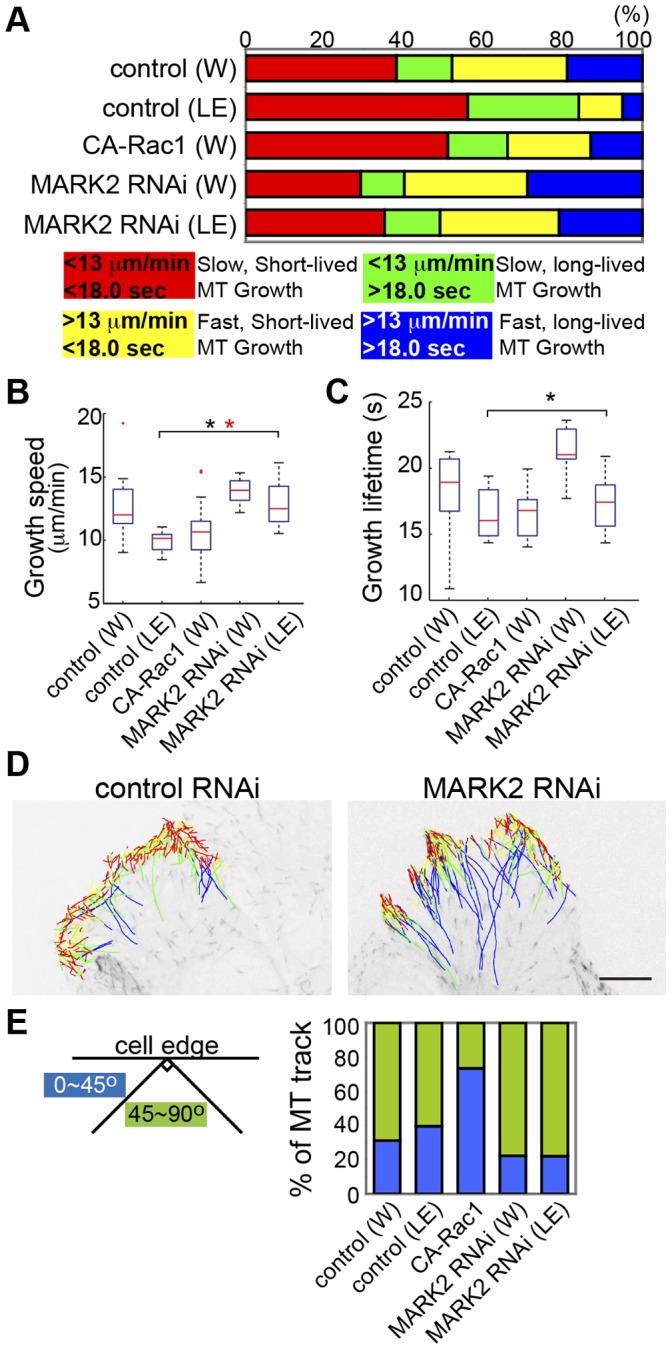
MARK2 is required for leading edge MT dynamics of directed migrating cells. (A) Above: Proportion of MT growth excursions in each subpopulation in control shRNA vector-transfected whole cells (W), expression of CA-Rac1, MARK2 shRNA treatment or from within 5 µm from the leading edge of wound edge cells with MARK2 shRNA (MARK2 RNAi (LE)) or control shRNA (control (LE)) treatment. Below: Color key showing MT growth speed and growth excursion lifetime ranges for subpopulations. Box-plots of speed (B) and lifetime (C) of MT growth excursions, conditions as in A. (*, p<0.001, Kolmogorov-Smirnov, *, p<0.05, Students). (D) mKO-EB3 tracks from 2 min time-lapse movies (frame rate = 3 s) of cells at the edge of a scratch-wound, colored according to the key in A overlaid on images of mKO-EB3 (inverted contrast) with MARK2 shRNA (MARK2 RNAi) or control shRNA (control RNAi) treatment. Only MT growth tracks that come within 5 µm of the leading edge are shown. Bar, 10 µm. (E) Percentage of MT growth tracks within 5 µm from the leading edge whose angle relative to the cell edge is between 0–45° (blue) or between 45–90° (green), conditions as in A.

To resolve these apparently disparate results, we sought to determine if MARK2 specifically functions in spatial organization of MT growth in polarized cells undergoing directed migration in a scratch wound assay, where Rac1 activity has been shown to be polarized towards the leading edge in other cell types [11]. We analyzed MT growth in polarized cells migrating at a wound edge, comparing MT growth parameters averaged from whole cells (W) to MT growth parameters from within 5 µm of the leading edge (LE). In control shRNA treated-wound-edge cells, MTs in the leading edge exhibited more slow and short-lived growth excursions that were more often parallel to the cell edge compared to MTs throughout the whole cell, typical of pioneer MTs and similar to the effects of CA-Rac1 on MT growth (Fig. 6A–E, Tables S1, S2, S7, Video S3). shRNA-mediated MARK2 depletion in wound-edge cells homogenized differences in MT growth speed between MTs at the leading edge and throughout the whole cell, increased MT growth lifetime in both the leading edge and whole cell, and reduced the proportion of MTs growing parallel to the cell edge compared to controls or cells expressing CA-Rac1 (Fig. 6A–E, Tables S1, S2, S7, Video S3). Thus, MARK2 is required for polarizing MT growth dynamics to form pioneer MTs that exhibit slow, short-lived growth parallel to leading lamellipodia in polarized, directionally migrating cells.

**Figure 7:**
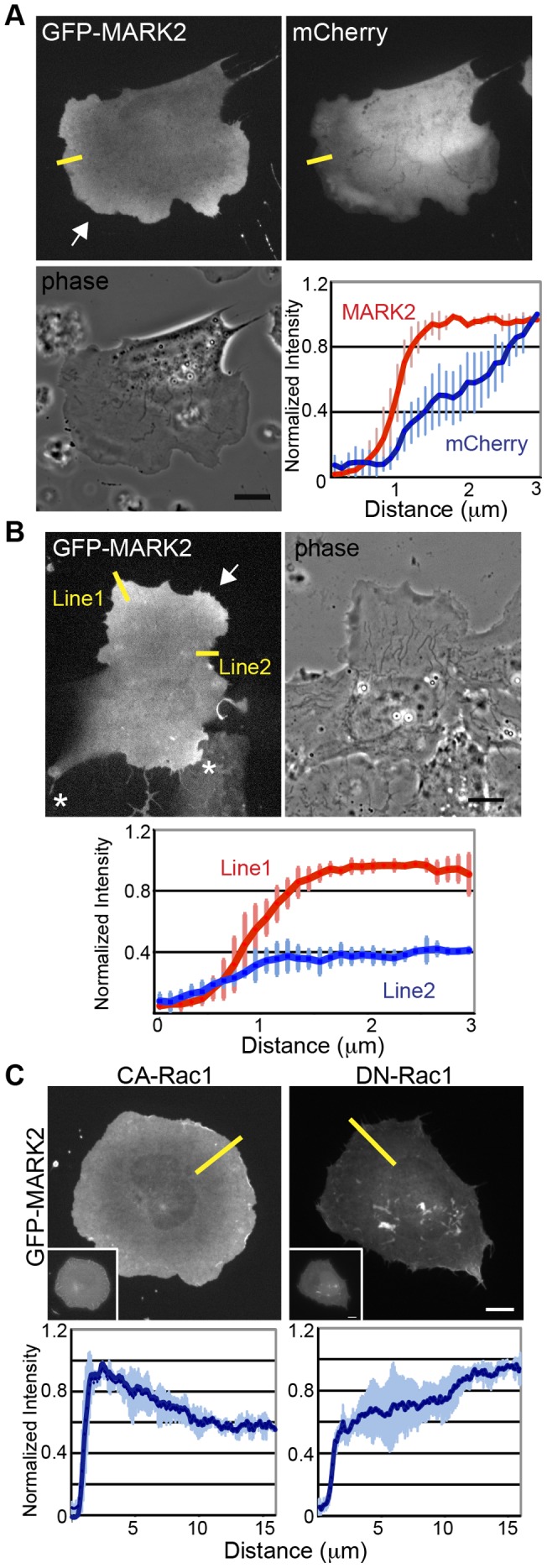
Accumulation of MARK2 in the leading edge depends on Rac1 activity. (A) Fluorescence images of GFP-MARK2 (upper left), mCherry-C1 (upper right) and phase image (lower left) in a non-polarized cell. Line scans (lower right) show the average normalized (to maximal) fluorescent intensity of three different cells expressing GFP-MARK2 (red) and mCherry-C1 (blue) along lines places similarly as those shown in the upper panel. Arrow represents cell protrusion. (B) Fluorescence image of GFP-MARK2 (upper left), and phase image (upper right) in migrating cell at a wound edge. Arrow: cell protrusions, Asterisks: cell-cell junctions. Line scans (lower panel) show average normalized (to maximal) fluorescent intensity of lines in three different cells expressing GFP-MARK2 along protrusion area (Lines 1) or non-protrusion area (Line 2). (C) Fluorescence images of GFP-MARK2 (upper left and upper right) in cell expressing BFP-CA-Rac1 (inset upper left) or BFP-DN-Rac1 (inset upper right). Insets show the BFP fluorescence images. Line scans indicate average normalized (to maximal) fluorescent intensity of three different cells expressing GFP-MARK2 along lines placed similarly to those in in CA-Rac1 (left) or DN-Rac1 (right). Bars, 10 µm.

We then sought to determine whether localization of MARK2 is polarized in migrating cells and/or controlled by Rac1 activity (Fig. 7). Unfortunately, available antibodies to MARK2 were not effective for immunofluorescnce under conditions required to preserve MT structure in fixed U2-OS cells. Comparing mCherry fluorescence as a volume marker to expressed GFP-MARK2 showed that GFP-MARK2 accumulated in protrusive regions of non-polarized cells (Fig. 7A). In migrating cells at a wound edge GFP-MARK2 localized in cell-cell junctions ([19], Fig. 7B) and was concentrated in the single leading protrusion facing the direction of migration (Fig. 7B). Co-expression of GFP-MARK2 and Rac1 mutants showed that CA-Rac1 induced concentration of GFP-MARK2 in lamellipodia around the entire cell periphery, and all peripheral localization was abolished in cells expressing DN-Rac1 (Fig. 7C). Thus, MARK2 localizes to the leading edge of directionally migrating cells in a Rac1-activity dependent-manner, suggesting MARK2 regulates MT organization in these regions.

**Figure 8:**
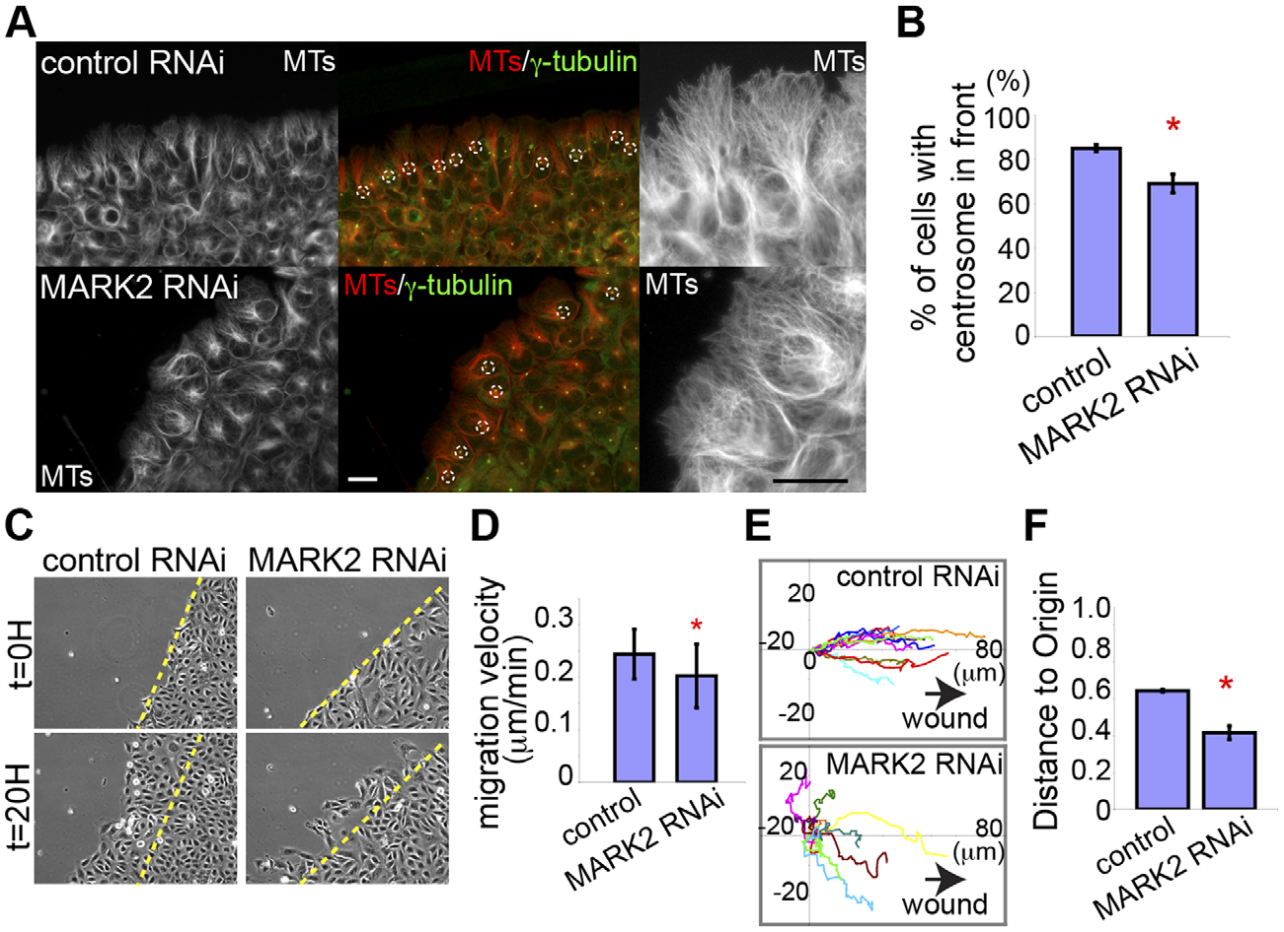
MARK2 is required for cell polarization and directional migration. (A) Immunostaining of MTs (left and right) and γ-tubulin (merge in center) in wound edge cells in non-targeting siRNA pool (top, control RNAi) and MARK2 siRNA treatment (bottom, MARK2 RNAi). Dotted circles, centrosome; bar, 10 µm. (B) Percentage of cells in the wound edge with centrosomes in front of the nucleus. In B, D, F *p<0.05, Student’s t-test. (C) Wound-healing assay in non-targeting siRNA pool (control RNAi; left) or MARK2 siRNA (MARK2 RNAi; right), time after wounding shown. Dashed line, position of wound edge at t = 0. Bar, 50 µm. (D) Average migration velocity of non-targeting siRNA pool (control) and MARK2 siRNA-treated (MARK2 RNAi) cells. (E) Rose plots of the position of nuclei over 5 hr (each cell track colored differently) for non-targeting siRNA pool-(above, control RNAi) or MARK2 siRNA-treated cells (below, MARK2 RNAi) at the edge of a wound. Arrows: open region of wound. (F) Distance from origin (position at time = 0) divided by total distance travelled over 5 hr for non-targeting siRNA pool and MARK2 siRNA treated cells.

We finally sought to determine the role of MARK2 in polarization and directional migration of U2-OS cells. To assay cell polarization, we examined effects of siRNA-mediated MARK2 depletion on centrosome orientation by immunolocalizing γ-tubulin in cells in a scratch wound assay. We utilized siRNA-mediated targeting in this experiment because of its increased transfection efficiency compared to shRNA. This allowed better determination of effects of MARK2 depletion on monolayer wound healing, which is coordinated by all cells in the monolayer, and may not be apparent with the lower level transfection afforded by shRNA methods. This showed that two hours after wounding, MARK2-depleted cells were inhibited in forward orientation of centrosomes, and MTs were less extended towards the leading edge compared to non-targeting siRNA treated-control cells (Fig. 8A, 8B). Analysis of time-lapse phase contrast movies showed that MARK2 depletion caused wound-edge cells to migrate significantly more slowly and with reduced directional persistence compared to control (Fig. 8C–F, Video S4, [42]). Together, these results suggest that MARK2 promotes directed cell migration via establishing polarity of the MT cytoskeleton and enhancing pioneer MT growth in the leading edge.

## Discussion

We utilized automated image analysis algorithms to screen for regulators of MT assembly dynamics induced downstream of Rac1 activation. Our results suggest that Op18/stathmin, the dynactin component p150*^glued^*, the +TIP proteins APC2 and EB1, the MT severing enzyme Spastin, and the kinase MARK2 may be potential downstream mediators of Rac1 signaling that regulate MT dynamics. Mechanistic links between Rac1 and APC or Op18 have previously been reported [13], [43], however the other factors identified here represent novel potential Rac1 targets. Thus, our results suggest that Rac1 acts as a master regulator of leading edge MT dynamics through multiple molecular pathways.

Our study represents the first time that parameters of MT growth dynamics have been used as the basis of a phenotypic screen, and was made possible by the development of automated image analysis programs [16], [17], [18]. This method allowed measurement of MT growth excursions throughout the cell, including short-lived, slow growth excursions that were not measurable by hand-tracking in previous studies [7], suggesting that activated Rac1 primarily promotes less persistent and slower polymerization of MTs, especially in the leading edge (Fig. 1C–E).

By further analyzing MT growth orientation in lamellipodia, we identified a single protein, MARK2, as required for Rac1 effects on both MT growth dynamics and orientation mediating pioneer MT formation. MARK2 is the mammalian homologue of the polarity protein PAR-1 which is thought to mediate polarity through effects on MTs [44]. However, this is the first report of MARK2/PAR1’s effects on polarity and MT dynamics being controlled by the Rac1 signaling cascade. We found that RNAis targeting any of the three MARK isoforms (1, 2, and 3) promoted fast MT assembly, suggesting that MARKs function to slow MT growth or destabilize MTs in situ, in agreement with previous studies [35]. Interestingly, depletion of MARK 1, 2 or 3 suppressed the slow, short-lived growth induced by CA-Rac1, but only depletion of MARK2 suppressed the effects of Rac1 on MT orientation in lamellipodia. Taken together, this suggests that all three MARK isoforms function downstream of Rac1 to regulate MT growth, but MARK2 additionally regulates Rac-1 mediated MT orientation.

The molecular mechanism by which Rac1 promotes MARK2-mediated regulation of MT growth dynamics and orientation in the leading edge is of interest. MARK2 is known to be activated by phosphorylation [23], and active MARK2 in turn phosphorylates MT-associated proteins (MAPs) such as tau, MAP2 and MAP4, which causes them to dissociate from MTs [23], [45], [46]. Notably, we found that RNAis targeting several MAPs including MAP4, MAP1B and MAP2, reduced the proportion of slow, short-lived MT growth excursions (Fig. 3A), similar to the effects of MARK2 depletion. Further study is needed to determine if Rac1 mediates local downstream inactivation of MARK2 in lamellipodia to reduce MAP phosphorylation and promote MAP-MT binding to locally stabilize MTs and drive pioneer MT growth. In addition, although we showed that Rac1 activity controls MARK2 localization and Rac1’s effects on MTs require MARK2, it would be of interest to additionally know if Rac1 controls MARK2’s kinase activity. MARK2 has been shown to interact with the Rac1 targets, GSK3β and a PAK kinase [41], [47], [48], and these in turn regulate Op18/stathmin and CLASP [13], [15]. This suggests that the MARK2, GSK3β, and PAK pathways of MT regulation could intersect downstream of Rac1.

The question of how MARK2 regulates MT orientation in lamellipodia is unclear. Since MT bending and growth parallel to the cell edge is thought to be driven by MT interactions with lamellipodial F-actin retrograde flow [5], [49], it is possible that MARK2’s effect on pioneer MT orientation are mediated by phosphoregulation of MAPs that bind both MTs and actin, as has been demonstrated for ACF7, APC and MAP2 [38], [50], [51], [52], [53], [54]. Alternatively, MARK2 may coordinately regulate MT and actin cytoskeletal dynamics through Pak kinases, which target both MT and actin binding proteins [55].

Our study builds on previous work showing that MARK kinases and their PAR1 homologues regulate cell polarity through downstream effects on MTs [44]. In a recent study of dendritic spines, it was found that that knockdown of the MARK2 homologue PAR-1 decreased the distance and duration of MT growth, as analyzed by tracking fluorescent +TIP proteins [56]. This contrasts our study in which MT growth persistence was increased in MARK2 knock-down cells (Figs. 3, 4 and 5). This suggests that MARK2 may utilize distinct effectors to control MT dynamics differently in each system. Notably, tracking of GFP-EB3 dynamics in *Drosophila* wing epidermis [57] and oocytes [58] revealed that the MARK2 homologue, Par-1, plays a crucial role in establishing a small bias in the orientation of growing MTs without a substantial change in overall MT distribution, resulting in asymmetric segregation of polarity proteins. In addition, similar to lamellipodia in migrating cells, MTs tend to bend and grow along the posterior cortex of *Drosophila* oocytes where Par-1 localizes, and Par-1 mutants fail to show these polarized MT growth excursions [58]. These data indicate that MARK2/PAR-1 has a conserved role in organizing MTs and regulating their growth and orientation in many systems.

Consistent with the role of MARK2 in MT reorganization, our results demonstrate that MARK2 is essential for cell polarization and directed cell migration. We found that MARK2-depleted cells in a wound edge have reduced pioneer MTs in the leading lamellipodia and show faster MT growth (Fig. 6). Indeed, depletion of MARK2 caused a failure in centrosome polarization towards the wound edge, and inhibited migration velocity and directionality (Fig. 8). MARK2/PAR-1 has also been shown to play a crucial role in directed migration in other cell types. For example, in the cerebral cortex, neurons depleted of MARK2 exhibit altered directed migration and reduced centrosome motility [59]. In addition, *par-1* null mutants in *Drosophila* border cells also fail in directed cell migration, showing abnormal protrusion in their front [60]. Therefore, MARK2/PAR-1 is a conserved key mediator for establishing cell polarity during directed migration, likely through regulating MT dynamics under the control of Rac1.

Taken together, our study shows the power of high-resolution, quantitative live cell imaging assays for refined screening of protein function, and identifies MARK2 as essential for linking Rac1 activation to polarization of MTs and their assembly dynamics critical to directed cell migration.

## Materials and Methods

### CDNA Expression Constructs

PCR products and products of restriction digests were purified by gel electrophoresis and extraction using the QIAquick™ gel extraction kit (QIAGEN, Valencia, CA, USA). Plasmid DNA was purified from overnight cultures using the QIAprep™ Spin Miniprep kit (QIAGEN). Restriction endonucleases were purchased from Life Technologies (Grand Island, NY, U.S.A.) or New England Biolabs (Ipswich, MA, U.S.A.). Sequencing was used to confirm the complete cDNA sequence (Florida State University Bioanalytical and Molecular Cloning DNA Sequencing Laboratory). mKusabira Orange (mKO) fused to EB3 was constructed using a N1 (Clontech-style) cloning vector. The fluorescent protein cDNA was PCR amplified (Phusion Flash; Finnzymes, Espoo, Finland) with a 5′ primer encoding an AgeI site and a 3′ primer encoding a NotI (N1) site. The purified and digested PCR products were ligated into a similarly digested EGFP-N1 cloning vector backbone (Clontech, Mountain View, CA, U.S.A.). Human EB3 cDNA (gift of Lynne Cassimeris, Lehigh University; NM_012326.2) was PCR amplified (Phusion Flash) with primers containing NheI and BamHI restriction enzyme sites and ligated into the mKO-N1 cloning vector to produce a fusion with EB3 attached to the N-terminus of mKO separated by a 6-amino acid linker. DNA for mammalian cell transfection was prepared using the Plasmid Maxi kit (QIAGEN). Proper localization was confirmed using widefield (Nikon 80i; TRITC filter set) and spinning disk microscopy (Olympus DSUIX81; TRITC filter set). RNAi-resistant MARK2 tagged with EGFP for rescue experiments was constructed by cloning a BglII-KpnI fragment of pENTR(tm)221-MARK2 (Invitrogen) into pEGFP-C1 (CLONTECH Laboratories, Inc., Mountain View, CA) and substituting sequence (5′-AATTATCGATAAAACG-3′) for sequence (5′-GATCATTGACAAGACT-3′, from GM Bioscience (Rockville, MD)). Human Rac1 G12V and T17N cDNA (Missouri S&T cDNA Resource Center, Rolla, MO) were PCR amplified with primers containing with a 5′ primer encoding an KpnI and a 3′ primer encoding a ApaI restriction enzyme sites and ligated into the BFP-tagC1 cloning vector to produce a fusion with Rac1 G12V or T17N attached to the C-terminus of BFP.

### Cell Culture and Transfection

U2-OS cells were obtained from American Type Culture Collection and maintained in McCoy’s 5A with 10% FBS and 1% penicillin-streptomycin (Invitrogen, Carlsbad, CA) at 37°C in 5% CO_2_. For live imaging, medium was supplemented with 25 mM HEPES pH = 7.2 and 30 units/ml Oxyrase. Transfections were performed with an nucleofector (solution kit V (Lonza, Walkersville, MD)). For stable expression of fluorescent EB3, transfected cells were selected for 2 weeks in media supplemented with 0.8 mg/ml G-418 (Invitrogen), and low level-expressing cells collected by FACS.

### Western Blot

Cells were lysed in 1X Laemmli sample buffer, separated on 4–12 or 4–20% SDS-PAGE gels (Invitrogen), transferred to immobilon-P membranes, and visualized by immunoblotting with enhanced chemiluminescence (GE Healthcare, Piscataway, NJ) with the following antibodies: Primary antibodies: Mouse anti-tubulin (1∶3,000, DM1A, Sigma-Aldrich, St. Louis, MO); rabbit anti-Par1 (1∶200, obtained from A. Suzuki, Yokohama-City University, Yokohama, Japan); mouse anti-GAPDH (1∶5,000, Cell Signaling, Danvers, MA); rabbit anti-Op18 (1∶1000, Agrisera, Vännäs, Sweden); rabbit anti-X-orbit antibody (1∶1000, obtained from R. Heald, UC Berkley, Berkley, CA); and mouse anti-p150*^glued^* (1∶1000, BD Biosciences, San Jose, CA). Secondary antibodies: HRP-conjugated anti-mouse or rabbit (1∶3,000, Jackson ImmunoResearch Laboratories, Inc, West Grove, PA).

### Immunofluorescence

Coverslips with bound cells were fixed in 4% paraformaldehyde and 0.5% Glutaraldehyde (Electron Microscopy Science) in PHEM buffer (60 mM PIPES, 27 mM HEPES, 10 mM EGTA, 8 mM MgSO_4_, pH7.0) for 20 minutes at room temperature, permeabilized with 0.1% Triton X-100 in PHEM for 5 min, free aldehydes were reacted with 0.1 M glycine for 5 min, cells were washed 3x for 5 min in PBS, and blocked in blocking solution (2% BSA IgG free and protease free in PBS; Jackson ImmunoResearch Laboratories, Inc.). Coverslips were incubated with mouse anti-γ tubulin (1∶200, AKT-15, Sigma-Aldrich) and/or rat anti-tubulin α (1∶300, YL 1/2, AbDSerotec, Oxford, UK) antibodies diluted in blocking solution overnight in a humid chamber at 4°C, washed 3X for 5 min in PBS, incubated with fluorophore-conjugated secondary antibodies (1∶250, Jackson ImmunoResearch Laboratories, Inc.) and Alexa Fluor 655 phalloidin (1∶400; Invitrogen) for 1 h, washed again, and mounted on a slide in mounting media (Dako).

### RNAi

U2-OS cells stably expressing mKO-EB3 were transfected by Amaxa nucleofector with SMARTpool (3 µg siRNA, Thermo Fisher Scientific, Lafayette, CO) or shRNA vector (1 µg DNA, Thermo Fisher Scientific), and cultured for 24 h at 37°C with 5% CO_2_. Control cells were either transfected with non-targeting pool siRNA (D-001810, Thermo Fisher Scientific) (Fig. 8) or control shRNA vector (Thermo Scientific,) (Figs. 1B–F, 2, 3, 4, 5, and 6). The next day, cells were transfected with BFP-tagC1-Rac1V12 and replated on coverslips, and experiments were performed 14–20 hours later to allow time for Rac1 expression. The following SMARTpools were used: APC (APC: L-003869), APC2 (APC2: L-009847), MACF1 (ACF7: L-013618), CKAP5 (XMAP215: L-006847), STIMN1 (Op18: L-005152), DCTN (p150*^glued^*: L-012874), CLIP1 (CLIP170: L-005294), CYLN2 (CLIP115: L-013511), MAP6 (STOP: L-026713), BPY2IP1 (MAP1S: L-016881), SPAST (Spastin: L-014070) and KATNA1 (katanin p60: L-005157, Thermo Scientific Open Biosystems). The following shRNA vectors were used: EB1 (pSuper-EB1, obtained from T. Wittmann, UCSF, San Francisco, CA), an shRNAmir-GFP library (Thermo Fisher Scientific Open Biosystems ) included shRNA vectors for CLASP2, DCTN2, DCX, MAP1A, MAP1B, MAP2, MAP4, MARK1, MARK2 and MARK3. For depletion of MARK2 in a scratch wound assay (Fig. 8), SMARTpools (L-004260, Thermo Fisher Scientific) were used. For depletion of MARK2 in Figure S1 and Tables S8, human par-1b shRNA vector (obtained from A. Suzuki, [40]) was used. Western blot analysis was performed to verify protein depletion for CLASP2, p150*^glued^* dynactin, Op18/stathmin, and MARK2.

### Microscopy

mKO-EB3 dynamics were imaged on a Yokogawa spinning disk confocal microscope system described in [18] using a 60×1.4 NA Plan Apo oil immersion objective lens (Nikon). Images of EB3 dynamics were generated using illumination from the 561 nm laser and were captured using a 500-ms exposure at 3 sec image intervals for 3 min on a Coolsnap HQ2 cooled CCD (Photometrics) operated in the 14-bit mode. Images of immunofluorescence, GFP-MARK2 and mCherry volume marker were acquired using the same microscope system, using the 561 or 488 nm lasers as appropriate. Microscope system automation was controlled with Metamorph software (Molecular Devices).

For wound healing assays, cells were grown to confluency and wounded 48 h after transfection by generating a longitudinal scratch using a razor blade. After a 2 h recovery at 37°C in 5% CO_2_, phase-contrast images were acquired at 5-min intervals for 20 h on the microscope described above using a 20×0.45 NA phase objective and an 0.52 NA LWD condenser using Metamorph’s Multi-dimensional Acquisition (MDA) software module.

### Image Analysis

MT growth dynamics were analyzed from EB3 time-lapse movies using plusTipTracker [16], [17] (http://lccb.hms.harvard.edu/software.html). For this study MT shrinkage or pause events were not estimated. Accordingly, we set the gap length to relatively short intervals to limit the gap closing mechanism in the software mostly to bridge short-term out-of-focus movements of the comets. The following parameter set was used for all movies in the dataset: maximum gap length, 5 frames; minimum track length, 3 frames; search radius range, 8–12 pixels; maximum forward angle, 30°; maximum backward angle, 10°; maximum shrinkage factor, 1.5; fluctuation radius, 1 pixel.

To categorize EB3 tracks based on MT growth speed and growth excursion lifetime, we utilized the tool within plusTipTracker called ‘Quadrant Scatter Plots.’ Briefly, the function generates a 2D scatter plot of speed versus lifetime with each point representing a single MT growth excursion defined by a single continuous EB3 track. Then, the points on the graph are divided into four subpopulations based on whether they were above or below the average growth speed (13 µm/min) and average growth lifetime (18 sec) of all EB3 tracks from all control cells analyzed in the study. These four subpopulations are coded by color, and a percentage bar showing the relative proportion of the subpopulations was generated. Colored tracks were overlaid on an inverted image of mKO-EB3 using the same color scheme to show how each subpopulation is distributed across the cell. To analyze the angle of MT growth excursions near the leading edge, EB3 tracks in these color overlays were analyzed within 5 µm from the edge of cell and their angle relative to the leading edge was categorized as parallel if the track was less than 45 degrees or perpendicular if more than 45 degrees.

For analysis of centrosome position in wound healing assays, images of fixed cells stained for MTs, γ-tubulin and DNA were aligned with the wound parallel to the image x axis and lines were plotted parallel to the image x axis to bisect each nucleus of cells at the wound edge. Centrosomes in front of the line were considered “oriented”.

Cell migration in wound healing assays was quantified by hand-tracking the nucleus in time-lapse phase-contrast image series using the “track points” function in MetaMorph software to determine instantaneous velocity and distance to origin divided by the total path length.

Statistical analysis was performed using the Analyze-It plug-in (Analyze-It Software Ltd.) for Excel (Microsoft) or Matlab. Box plots were used to represent the distributions of MT growth speed and lifetime measurements under different conditions: red lines represent median and boxes around them represent 25^th^ and 75^th^ percentile of the dataset; 98% of the data points are inside the whisker area and the residual outliers are represented by the red points. For analysis of MT growth speed and lifetimes, Kolmogorov-Smirnov test was used to analyze potential differences between the distributions of all measured MT growth excursions pooled from all cells under each condition, and Student’s t-test was used to analyze potential differences between the distributions of mean values for each cell under each condition. For centrosome position and cell migration velocity and directionality measurements, Student’s t-test was used to analyze potential differences between the distributions of mean values for each condition.

## Supporting Information

Figure S1 **shRNA targeting of a distinct sequence of MARK2 has similar effects on MT dynamics.** An shRNA vector with sequence targeting MARK2 (human par-1b) was obtained from A. Suzuki [40]. (A) Top: Proportion of MT growth excursions in subpopulations of CA-Rac1-transfected (CA-Rac1), CA-Rac1 and MARK2 shRNA-transfected (+CA-Rac1, MARK2 RNAi #2), or CA-Rac1, MARK2 shRNA and GFP-MARK2-transfected cells (+CA-Rac1, MARK2 RNAi #2+GFP-MARK2). Bottom: Color key showing MT growth speed and growth excursion lifetime ranges for subpopulations. Box-plots of speed (B) and lifetime (C) of MT growth excursions, conditions as in A. (*, p<0.001, Kolmogorov-Smirnov, *, p<0.05, Students).
(TIF)

Table S1 **Mean MT growth speed and growth excursion lifetimes.** Control cells were transfected with control shRNA vector; MARK2 shRNA vector was used for MARK2 RNAi. Results of analysis of mKO-EB3 time-lapse movies using PlusTipTracker software to measure MT growth dynamics. Data shown is depicted graphically in Fig. 1D, 1E; Fig. 4D, 4E; Fig. 5C, 5D; Fig. 6B, 6C.
(DOC)

Table S2 **Proportion of MT growth excursions in subpopulations grouped according to growth speed and growth excursion lifetime.** Control cells were transfected with control shRNA vector; MARK2 shRNA vector was used for MARK2 RNAi. Data shown is depicted graphically in Fig. 1C, Fig. 4C, Fig. 5B, Fig. 6A.
(DOC)

Table S3 **Mean MT growth speed and growth excursion lifetimes for cells treated with RNAis.** shRNA vectors were used for RNAi targeting of EB1, CLASP2, dynamitin, DCX, MAP1A, MAP1B, MAP2, MAP4, MARK1, MARK2 and MARK3. siRNA oligos were used for RNAi targeting of APC, APC2, ACF7, XMAP215, Op18, p150*^glued^*, CLIP115, CLIP170, STOP, MAP1S, Spastin and Katanin p60. Results of analysis of mKO-EB3 time-lapse movies using PlusTipTracker software to measure MT growth dynamics.
(DOC)

Table S4 **Proportion of MT growth excursions in subpopulations grouped according to growth speed and growth excursion lifetime for cells treated with RNAsi.** shRNA vectors were used for RNAi targeting of EB1, CLASP2, dynamitin, DCX, MAP1A, MAP1B, MAP2, MAP4, MARK1, MARK2 and MARK3. siRNA oligos were used for RNAi targeting of APC, APC2, ACF7, XMAP215, Op18, p150*^glued^*, CLIP115, CLIP170, STOP, MAP1S, Spastin and Katanin p60. Results of analysis of mKO-EB3 time-lapse movies using PlusTipTracker software to measure MT growth dynamics. Data shown is depicted graphically in Figure 2.
(DOC)

Table S5 **Mean MT growth speed and growth excursion lifetimes for cells expressing CA-Rac1 and treated with RNAis.** shRNA vectors were used for RNAi targeting of EB1, CLASP2, dynamitin, DCX, MAP1A, MAP1B, MAP2, MAP4, MARK1, MARK2 and MARK3. siRNA oligos were used for RNAi targeting of APC, APC2, ACF7, XMAP215, Op18, p150glued, CLIP115, CLIP170, STOP, MAP1S, Spastin and Katanin p60. Results of analysis of mKO-EB3 time-lapse movies using PlusTipTracker software to measure MT growth dynamics.
(DOC)

Table S6 **Proportion of MT growth excursions in subpopulations grouped according to growth speed and growth excursion lifetime for cells expressing CA-Rac1 and treated with RNAis.** shRNA vectors were used for RNAi targeting of EB1, CLASP2, dynamitin, DCX, MAP1A, MAP1B, MAP2, MAP4, MARK1, MARK2 and MARK3. siRNA oligos were used for RNAi targeting of APC, APC2, ACF7, XMAP215, Op18, p150*^glued^*, CLIP115, CLIP170, STOP, MAP1S, Spastin and Katanin p60. Results of analysis of mKO-EB3 time-lapse movies using PlusTipTracker software to measure MT growth dynamics. Data shown is depicted graphically in Figure 3A.
(DOC)

Table S7 **Orientation of MT growth at the leading edge.** shRNA vectors were used for control, and RNAi targeting of EB1 and MARK2. siRNA oligos were used for RNAi targeting of APC2, Op18, p150*^glued^* and Spastin. mKO-EB3 tracks were overlaid on images of mKO-EB3 using PlusTipTracker software and the orientation of tracks within 5 µm from the leading edge was classified according to their angle relative to the cell edge. Data shown is depicted graphically in Figures. (Fig. 1G, Fig. 3C, Fig. 4G, Fig. 5F, Fig. 6E).
(DOC)

Table S8 **Mean MT growth speed and growth excursion lifetimes in Figure S1.** MARK2 shRNA #2 vector was used for MARK2 RNAi. Results of analysis of mKO-EB3 time-lapse movies using PlusTipTracker software to measure MT growth dynamics. Data shown is depicted graphically in Figure S1B and S1C.
(DOC)

Video S1 **EB3 dynamics in U2-OS cells expressing Rac1 mutants.** Spinning disk confocal fluorescence time-lapse image sequences of cells expressing mKO-EB3 to mark growing MT plus ends in cells expressing control non-targeting vector (WT, left), CA-Rac1 (middle), and DN-Rac1 (right). The same cells are shown in Figure 1F. Bar  = 10 µm, elapsed time in min: sec shown.
(MOV)

Video S2 **EB3 dynamics in U2-OS cells treated with shRNAs targeting MARK2.** Spinning disk confocal fluorescence time-lapse image sequences of cells expressing mKO-EB3 to mark growing MT plus ends in cells expressing shRNA targeting MARK2 (left), CA-Rac1 and shRNA targeting MARK2 (middle) or expressing CA-Rac1, shRNA targeting MARK2, and RNAi-resistant GFP-MARK2 (right). The same cells are shown in Figure 4F and Figure 5E. Bar  = 10 µm, elapsed time in min: sec.
(MOV)

Video S3 **EB3 dynamics in control and MARK2 depleted cells in a monolayer wound edge.** Spinning disk confocal fluorescence time-lapse image sequences of cells expressing mKO-EB3 two hours after scratch-wounding a cell monolayer. Cells treated with control shRNA (left), or MARK2 shRNA (right) at the edge of a monolayer wound. The same cells are shown in Figure 6D, bar  = 10 µm, elapsed time in min: sec.
(MOV)

Video S4 **Effects of MARK2 depletion on directed cell migration**. Phase contrast time-lapse image series of U2-OS cells treated with non-targeting siRNA pool (left) or MARK2 siRNA (right). The same cells are shown in Figure 8C, bar  = 50 µm, elapsed time after scratch-wounding shown in hr: min.
(MOV)
