# Supplementary figures and images for: Automated Screening of Microtubule Growth Dynamics Identifies MARK2 as a Regulator of Leading Edge Microtubules Downstream of Rac1 in Migrating Cells
Source: PLoS One. 2012 Jul 24;7(7):e41413. doi: 10.1371/journal.pone.0041413 (PMC3404095; doi:10.1371/journal.pone.0041413)

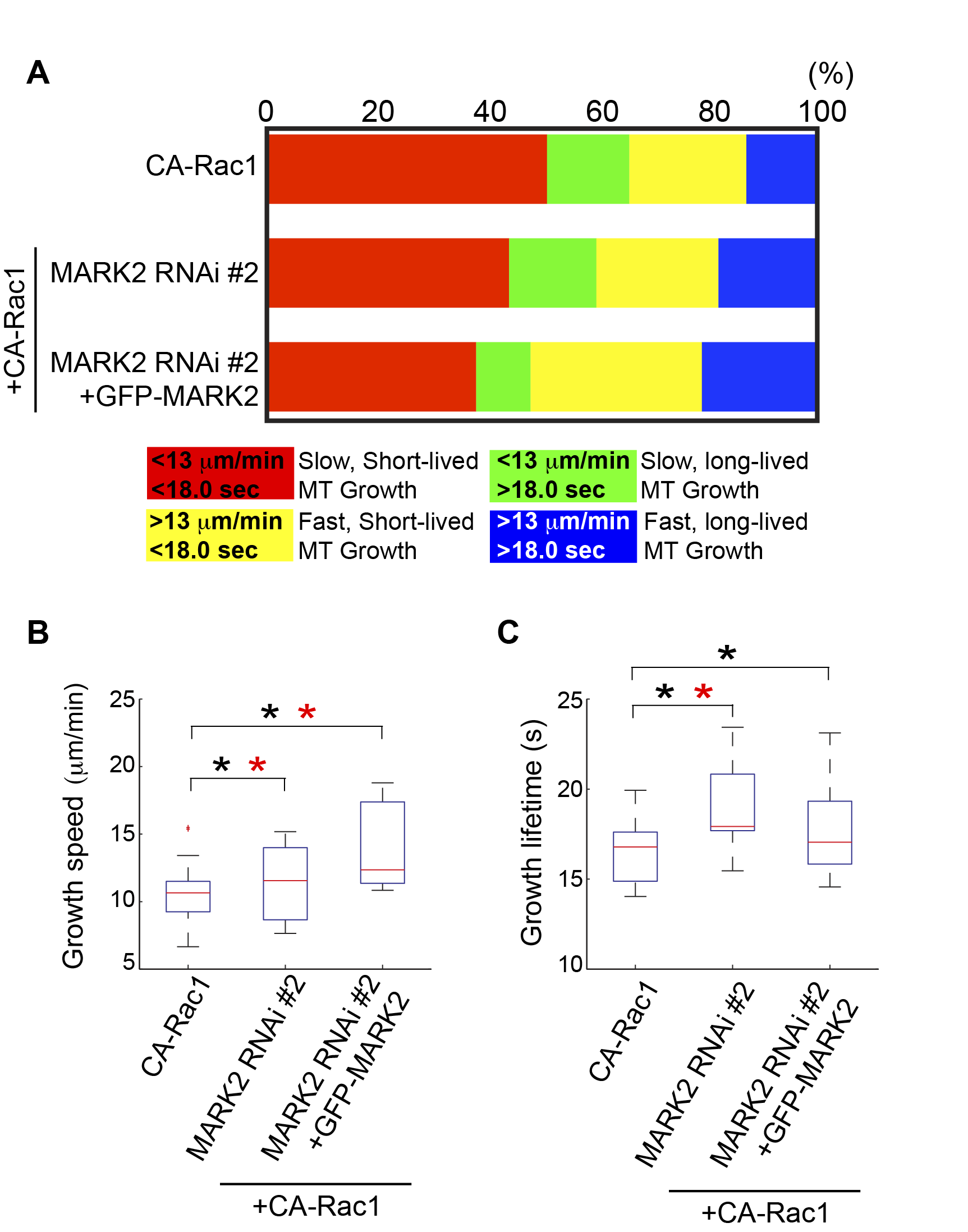

Supplement: Figure S1 — shRNA targeting of a distinct sequence of MARK2 has similar effects on MT dynamics. An shRNA vector with sequence targeting MARK2 (human par-1b) was obtained from A. Suzuki [40]. (A) Top: Proportion of MT growth excursions in subpopulations of CA-Rac1-transfected (CA-Rac1), CA-Rac1 and MARK2 shRNA-transfected (+CA-Rac1, MARK2 RNAi #2), or CA-Rac1, MARK2 shRNA and GFP-MARK2-transfected cells (+CA-Rac1, MARK2 RNAi #2+GFP-MARK2). Bottom: Color key showing MT growth speed and growth excursion lifetime ranges for subpopulations. Box-plots of speed (B) and lifetime (C) of MT growth excursions, conditions as in A. (*, p<0.001, Kolmogorov-Smirnov, *, p<0.05, Students). (TIF) [file pone.0041413.s001.tif]
