# Supplementary material for: Automated Screening of Microtubule Growth Dynamics Identifies MARK2 as a Regulator of Leading Edge Microtubules Downstream of Rac1 in Migrating Cells
Source: PLoS One. 2012 Jul 24;7(7):e41413. doi: 10.1371/journal.pone.0041413 (PMC3404095; doi:10.1371/journal.pone.0041413)
Supplement: Table S1 — Mean MT growth speed and growth excursion lifetimes. Control cells were transfected with control shRNA vector; MARK2 shRNA vector was used for MARK2 RNAi. Results of analysis of mKO-EB3 time-lapse movies using PlusTipTracker software to measure MT growth dynamics. Data shown is depicted graphically in Fig. 1D, 1E; Fig. 4D, 4E; Fig. 5C, 5D; Fig. 6B, 6C. (DOC) [file pone.0041413.s002.doc]

| condition | Speed (μm/min)(mean +/- SEM) | Lifetime (s)(mean +/- SEM) | n= growth excursions | n=number of cells |
| --- | --- | --- | --- | --- |
| control | 12.71± 0.034 | 18.76± 0.091 | 30435 | 13 |
| CA-Rac1 | 10.70± 0.025 | 16.99± 0.068 | 48151 | 21 |
| DN-Rac1 | 14.77± 0.037 | 16.42± 0.080 | 26760 | 13 |
| MARK2 RNAi | 14.20± 0.044 | 21.85± 0.162 | 12859 | 6 |
| MARK2 RNAi+MARK2 GFP | 16.18± 0.057 | 18.11± 0.137 | 10939 | 6 |
| CA-Rac1+MARK2 RNAi | 12.64± 0.047 | 20.57± 0.156 | 13591 | 6 |
| CA-Rac1+MARK2RNAi+MARK2 GFP | 16.17± 0.050 | 18.41± 0.119 | 15907 | 7 |
| Leading edge of wound  (control) | 9.77± 0.063 | 17.97± 0.248 | 3181 | 10 |
| Leading edge of wound (MARK2 RNAi) | 13.05± 0.072 | 19.54± 0.233 | 4500 | 10 |
