# Supplementary material for: Automated Screening of Microtubule Growth Dynamics Identifies MARK2 as a Regulator of Leading Edge Microtubules Downstream of Rac1 in Migrating Cells
Source: PLoS One. 2012 Jul 24;7(7):e41413. doi: 10.1371/journal.pone.0041413 (PMC3404095; doi:10.1371/journal.pone.0041413)
Supplement: Table S2 — Proportion of MT growth excursions in subpopulations grouped according to growth speed and growth excursion lifetime. Control cells were transfected with control shRNA vector; MARK2 shRNA vector was used for MARK2 RNAi. Data shown is depicted graphically in Fig. 1C, Fig. 4C, Fig. 5B, Fig. 6A. (DOC) [file pone.0041413.s003.doc]

| condition | % slow,short-lived  (<13μm/min, <18s) | % slow,  long-lived  (<13μm/min, >18s) | % fast,  short-lived  (>13μm/min, <18s) | % fast,  long-lived  (>13μm/min, >18s) |
| --- | --- | --- | --- | --- |
| control | 38 | 14 | 29 | 19 |
| CA-Rac1 | 51 | 15 | 21 | 13 |
| DN-Rac1 | 33 | 6 | 40 | 21 |
| MARK2 RNAi | 29 | 11 | 31 | 29 |
| MARK2 RNAi+MARK2 GFP | 24 | 3 | 44 | 29 |
| CA-Rac1+MARK2 RNAi | 40 | 13 | 25 | 22 |
| CA-Rac1+MARK2RNAi+MARK2 GFP | 27 | 5 | 41 | 27 |
| Leading edge of wound (control) | 56 | 28 | 11 | 5 |
| Leading edge of wound (MARK2 RNAi) | 35 | 14 | 30 | 21 |
