# Supplementary material for: Automated Screening of Microtubule Growth Dynamics Identifies MARK2 as a Regulator of Leading Edge Microtubules Downstream of Rac1 in Migrating Cells
Source: PLoS One. 2012 Jul 24;7(7):e41413. doi: 10.1371/journal.pone.0041413 (PMC3404095; doi:10.1371/journal.pone.0041413)
Supplement: Table S3 — Mean MT growth speed and growth excursion lifetimes for cells treated with RNAis. shRNA vectors were used for RNAi targeting of EB1, CLASP2, dynamitin, DCX, MAP1A, MAP1B, MAP2, MAP4, MARK1, MARK2 and MARK3. siRNA oligos were used for RNAi targeting of APC, APC2, ACF7, XMAP215, Op18, p150glued, CLIP115, CLIP170, STOP, MAP1S, Spastin and Katanin p60. Results of analysis of mKO-EB3 time-lapse movies using PlusTipTracker software to measure MT growth dynamics. (DOC) [file pone.0041413.s004.doc]

| condition (RNAi (kd)) | Speed (μm/min) (mean +/- SEM) | Lifetime (s) (mean +/- SEM) | n= growth excursions | n=number of cells |
| --- | --- | --- | --- | --- |
| CLIP115/170kd | 8.11±0.040 | 17.63±0.144 | 10880 | 9 |
| Doblecortin kd | 9.10±0.038 | 18.35±0.131 | 15477 | 9 |
| APC2 kd | 9.40±0.031 | 19.31±0.124 | 17795 | 10 |
| XMAP215 kd | 9.43±0.036 | 12.00±0.064 | 21614 | 5 |
| MAP1S kd | 10.22±0.040 | 17.40±0.109 | 16988 | 15 |
| STOP kd | 10.34±0.046 | 17.66±0.135 | 11629 | 10 |
| Spastin kd | 11.36±0.046 | 17.40±0.124 | 13742 | 10 |
| MAP1A kd | 11.58±0.044 | 17.50±0.121 | 14132 | 10 |
| EB1 kd | 11.79±0.039 | 17.99±0.113 | 18178 | 10 |
| MAP1B kd | 11.84±0.039 | 21.84±0.153 | 16315 | 7 |
| p150*^glued^* kd | 11.93±0.048 | 16.92±0.109 | 16185 | 13 |
| APC kd | 12.84±0.039 | 17.23±0.098 | 20394 | 13 |
| MAP4 kd | 13.82±0.053 | 14.85±0.105 | 13445 | 11 |
| MARK2 kd | 14.21±0.043 | 21.85±0.160 | 12859 | 6 |
| Dynamitin kd | 14.73±0.058 | 16.79±0.130 | 11204 | 7 |
| MAP2 kd | 14.95±0.047 | 17.08±0.109 | 15699 | 10 |
| MARK1 kd | 15.93±0.045 | 17.67±0.112 | 15451 | 8 |
| MARK3 kd | 16.27±0.042 | 17.97±0.100 | 21900 | 14 |
| Op18 kd | 16.64±0.046 | 17.51±0.106 | 17583 | 11 |
| Katanin p60 kd | 17.69±0.055 | 17.35±0.107 | 16355 | 9 |
| ACF7 kd | 17.96±0.073 | 15.92±0.135 | 8091 | 4 |
| CLASP2 kd | 18.93±0.067 | 17.22±0.135 | 9867 | 5 |
