# Supplementary material for: Automated Screening of Microtubule Growth Dynamics Identifies MARK2 as a Regulator of Leading Edge Microtubules Downstream of Rac1 in Migrating Cells
Source: PLoS One. 2012 Jul 24;7(7):e41413. doi: 10.1371/journal.pone.0041413 (PMC3404095; doi:10.1371/journal.pone.0041413)
Supplement: Table S4 — Proportion of MT growth excursions in subpopulations grouped according to growth speed and growth excursion lifetime for cells treated with RNAsi. shRNA vectors were used for RNAi targeting of EB1, CLASP2, dynamitin, DCX, MAP1A, MAP1B, MAP2, MAP4, MARK1, MARK2 and MARK3. siRNA oligos were used for RNAi targeting of APC, APC2, ACF7, XMAP215, Op18, p150glued, CLIP115, CLIP170, STOP, MAP1S, Spastin and Katanin p60. Results of analysis of mKO-EB3 time-lapse movies using PlusTipTracker software to measure MT growth dynamics. Data shown is depicted graphically in Figure 2. (DOC) [file pone.0041413.s005.doc]

| condition  (RNAi (kd)) | % slow,short-lived  (<13 μm/min, <18s) | % slow,long-lived  (<13 μm/min, >18s) | % fast,short-lived  (>13 μm/min, <18s) | % fast,long-lived  (>13 μm /min, >18s) |
| --- | --- | --- | --- | --- |
| CLIP115/170 kd | 64 | 26 | 7 | 3 |
| XMAP215 kd | 64 | 10 | 21 | 5 |
| Doublecortin kd | 57 | 23 | 12 | 8 |
| APC2 kd | 56 | 27 | 11 | 6 |
| MAP1S kd | 53 | 17 | 18 | 12 |
| STOP kd | 52 | 18 | 18 | 12 |
| Spastin kd | 47 | 9 | 33 | 21 |
| MAP1A kd | 46 | 14 | 25 | 15 |
| p150*^glued^* kd | 44 | 15 | 27 | 14 |
| EB1 kd | 44 | 13 | 26 | 17 |
| MAP1B kd | 41 | 18 | 22 | 19 |
| APC kd | 39 | 11 | 32 | 18 |
| MAP4 kd | 37 | 41 | 7 | 15 |
| Dynamitin kd | 31 | 5 | 42 | 22 |
| MAP2 kd | 30 | 6 | 41 | 23 |
| MARK2 kd | 29 | 11 | 31 | 29 |
| MARK1 kd | 24 | 5 | 45 | 26 |
| MARK3 kd | 24 | 5 | 45 | 26 |
| Op18 kd | 23 | 5 | 47 | 25 |
| Katanin p60 kd | 23 | 3 | 46 | 26 |
| ACF7 kd | 20 | 2 | 54 | 24 |
| CLASP2 kd | 17 | 2 | 53 | 28 |
