# Supplementary material for: Automated Screening of Microtubule Growth Dynamics Identifies MARK2 as a Regulator of Leading Edge Microtubules Downstream of Rac1 in Migrating Cells
Source: PLoS One. 2012 Jul 24;7(7):e41413. doi: 10.1371/journal.pone.0041413 (PMC3404095; doi:10.1371/journal.pone.0041413)
Supplement: Table S5 — Mean MT growth speed and growth excursion lifetimes for cells expressing CA-Rac1 and treated with RNAis. shRNA vectors were used for RNAi targeting of EB1, CLASP2, dynamitin, DCX, MAP1A, MAP1B, MAP2, MAP4, MARK1, MARK2 and MARK3. siRNA oligos were used for RNAi targeting of APC, APC2, ACF7, XMAP215, Op18, p150glued, CLIP115, CLIP170, STOP, MAP1S, Spastin and Katanin p60. Results of analysis of mKO-EB3 time-lapse movies using PlusTipTracker software to measure MT growth dynamics. (DOC) [file pone.0041413.s006.doc]

| condition (RNAi (kd)) | Speed (μm /min) (mean +/- SEM) | Lifetime (s) (mean +/- SEM) | n= growth excursions | n=number of cells |
| --- | --- | --- | --- | --- |
| MAP1S kd | 5.94±0.036 | 18.32±0.155 | 10909 | 7 |
| XMAP215 kd | 6.81±0.042 | 14.23±0.126 | 11776 | 6 |
| APC kd | 8.26±0.035 | 17.28±0.123 | 17212 | 9 |
| MARK1 kd | 8.81±0.060 | 16.44±0.137 | 10949 | 5 |
| STOP kd | 8.97±0.034 | 18.82±0.124 | 19615 | 10 |
| MAP1A kd | 9.24±0.050 | 16.36±0.140 | 10293 | 5 |
| Doublecortin kd | 9.34±0.032 | 17.34±0.098 | 24526 | 10 |
| MARK3 kd | 9.62±0.039 | 13.78±0.083 | 24451 | 13 |
| CLIP115/170 kd | 10.09±0.038 | 19.30±0.129 | 18788 | 10 |
| Dynamitin kd | 11.24±0.039 | 20.00±0.134 | 18633 | 10 |
| MAP4 kd | 11.79±0.045 | 18.45±0.129 | 15843 | 8 |
| p150*^glued^* kd | 12.34±0.040 | 17.90±0.104 | 21258 | 10 |
| APC2 kd | 12.77±0.038 | 19.33±0.113 | 21637 | 9 |
| MARK2 kd | 12.64±0.040 | 20.57±0.119 | 13591 | 6 |
| EB1 kd | 13.47±0.034 | 20.06±0.105 | 27725 | 10 |
| Spastin kd | 13.48±0.040 | 17.41±0.096 | 22008 | 10 |
| Op18 kd | 14.03±0.049 | 17.99±0.110 | 18527 | 10 |
| MAP1B kd | 15.32±0.036 | 17.67±0.086 | 29596 | 10 |
| Katanin p60 kd | 15.35±0.040 | 17.93±0.091 | 28015 | 10 |
| MAP2 kd | 15.48±0.041 | 17.83±0.095 | 23088 | 10 |
| ACF7 kd | 16.86±0.038 | 18.08±0.085 | 29675 | 13 |
| CLASP2 kd | 18.01±0.048 | 20.16±0.108 | 23793 | 6 |
