# Supplementary material for: Automated Screening of Microtubule Growth Dynamics Identifies MARK2 as a Regulator of Leading Edge Microtubules Downstream of Rac1 in Migrating Cells
Source: PLoS One. 2012 Jul 24;7(7):e41413. doi: 10.1371/journal.pone.0041413 (PMC3404095; doi:10.1371/journal.pone.0041413)
Supplement: Table S6 — Proportion of MT growth excursions in subpopulations grouped according to growth speed and growth excursion lifetime for cells expressing CA-Rac1 and treated with RNAis. shRNA vectors were used for RNAi targeting of EB1, CLASP2, dynamitin, DCX, MAP1A, MAP1B, MAP2, MAP4, MARK1, MARK2 and MARK3. siRNA oligos were used for RNAi targeting of APC, APC2, ACF7, XMAP215, Op18, p150glued, CLIP115, CLIP170, STOP, MAP1S, Spastin and Katanin p60. Results of analysis of mKO-EB3 time-lapse movies using PlusTipTracker software to measure MT growth dynamics. Data shown is depicted graphically in Figure 3A. (DOC) [file pone.0041413.s007.doc]

| condition  (RNAi (kd)) | % slow,short-lived  (<13 μm/min, <18s) | % slow,long-lived  (<13 μm/min, >18s) | % fast,short-lived  (>13 μm/min, <18s) | % fast,long-lived  (>13 μm/min, >18s) |
| --- | --- | --- | --- | --- |
| XMAP215 kd | 73 | 16 | 9 | 2 |
| MAP1S kd | 66 | 29 | 3 | 2 |
| APC kd | 63 | 21 | 10 | 6 |
| MARK3 kd | 63 | 12 | 16 | 9 |
| MAP1A kd | 60 | 16 | 14 | 10 |
| MARK1 kd | 60 | 15 | 14 | 11 |
| STOP kd | 58 | 22 | 12 | 8 |
| Doublecortin kd | 57 | 19 | 14 | 10 |
| CLIP115/170 kd | 53 | 19 | 16 | 12 |
| Dynamitin kd | 46 | 14 | 20 | 20 |
| MAP4 kd | 46 | 14 | 23 | 17 |
| p150*^glued^* kd | 43 | 11 | 27 | 19 |
| MARK2 kd | 40 | 13 | 25 | 22 |
| APC2 kd | 39 | 10 | 27 | 24 |
| Spastin kd | 37 | 9 | 33 | 21 |
| EB1 kd | 36 | 12 | 29 | 23 |
| Op18 kd | 36 | 10 | 33 | 21 |
| katanin p60 kd | 31 | 7 | 39 | 23 |
| MAP1B kd | 31 | 7 | 39 | 23 |
| MAP2 kd | 30 | 6 | 39 | 25 |
| ACF7 kd | 24 | 4 | 45 | 27 |
| CLASP2 kd | 21 | 4 | 42 | 33 |
