# Supplementary material for: Automated Screening of Microtubule Growth Dynamics Identifies MARK2 as a Regulator of Leading Edge Microtubules Downstream of Rac1 in Migrating Cells
Source: PLoS One. 2012 Jul 24;7(7):e41413. doi: 10.1371/journal.pone.0041413 (PMC3404095; doi:10.1371/journal.pone.0041413)
Supplement: Table S7 — Orientation of MT growth at the leading edge. shRNA vectors were used for control, and RNAi targeting of EB1 and MARK2. siRNA oligos were used for RNAi targeting of APC2, Op18, p150glued and Spastin. mKO-EB3 tracks were overlaid on images of mKO-EB3 using PlusTipTracker software and the orientation of tracks within 5 µm from the leading edge was classified according to their angle relative to the cell edge. Data shown is depicted graphically in Figures. (Fig. 1G, Fig. 3C, Fig. 4G, Fig. 5F, Fig. 6E). (DOC) [file pone.0041413.s008.doc]

| condition | parallel (0-45^o^) | perpendicular (45-90^o^) | n=growth excursions | n=number of cells |
| --- | --- | --- | --- | --- |
| control | 31.1 | 68.9 | 765 | 10 |
| CA-Rac1 | 73.4 | 26.6 | 738 | 10 |
| DN-Rac1 | 23.3 | 76.7 | 733 | 7 |
| CA-Rac1+p150*^glued^* kd | 75.3 | 24.7 | 1080 | 10 |
| CA-Rac1+MARK2 kd | 37.3 | 62.7 | 783 | 11 |
| CA-Rac1+APC2 kd | 70.0 | 30.0 | 747 | 9 |
| CA-Rac1+Spastin kd | 65.7 | 34.3 | 764 | 9 |
| CA-Rac1+EB1 kd | 72.2 | 27.8 | 880 | 10 |
| CA-Rac1+Op18 kd | 66.9 | 33.1 | 812 | 10 |
| MARK2 kd | 22.2 | 77.8 | 751 | 8 |
| MARK2 kd+GFP-MARK2 | 21.7 | 78.3 | 891 | 6 |
| control (LE) | 39.5 | 60.5 | 679 | 10 |
| MARK2 kd (LE) | 22.0 | 78.0 | 990 | 10 |
