# Supplementary material for: Automated Screening of Microtubule Growth Dynamics Identifies MARK2 as a Regulator of Leading Edge Microtubules Downstream of Rac1 in Migrating Cells
Source: PLoS One. 2012 Jul 24;7(7):e41413. doi: 10.1371/journal.pone.0041413 (PMC3404095; doi:10.1371/journal.pone.0041413)
Supplement: Table S8 — Mean MT growth speed and growth excursion lifetimes in Figure S1. MARK2 shRNA #2 vector was used for MARK2 RNAi. Results of analysis of mKO-EB3 time-lapse movies using PlusTipTracker software to measure MT growth dynamics. Data shown is depicted graphically in Figure S1B and S1C. (DOC) [file pone.0041413.s009.doc]

| **condition** | **Speed (μm/min)(mean +/- SEM)** | **Lifetime (s)(mean +/- SEM)** | **n= growth excursions** | **n=number of cells** |
| --- | --- | --- | --- | --- |
| **CA-Rac1** | 10.70± 0.025 | 16.99± 0.068 | 48151 | 21 |
| **CA-Rac1+MARK2 RNAi #2** | 11.53± 0.037 | 19.98± 0.132 | 22718 | 9 |
| **CA-Rac1+MARK2RNAi #2**  **+MARK2 GFP** | 13.93± 0.065 | 18.11± 0.167 | 11669 | 5 |
